# A Functional MRI Paradigm for Efficient Mapping of Memory Encoding Across Sensory Conditions

**DOI:** 10.3389/fnhum.2020.591721

**Published:** 2021-01-21

**Authors:** Meta M. Boenniger, Kersten Diers, Sibylle C. Herholz, Mohammad Shahid, Tony Stöcker, Monique M. B. Breteler, Willem Huijbers

**Affiliations:** ^1^Population Health Sciences, German Center for Neurodegenerative Diseases (DZNE), Bonn, Germany; ^2^Image Analysis Group, German Center for Neurodegenerative Diseases (DZNE), Bonn, Germany; ^3^MR Physics, German Center for Neurodegenerative Diseases (DZNE), Bonn, Germany; ^4^Institute for Medical Biometry, Informatics and Epidemiology (IMBIE), Faculty of Medicine, University of Bonn, Bonn, Germany; ^5^Department of Electrical Engineering, Eindhoven University of Technology, Eindhoven, Netherlands

**Keywords:** memory encoding, sensory encoding, functional magnetic resonance imaging, epidemiologic research design, auditory cortex, visual cortex, hippocampus, parietal lobe

## Abstract

We introduce a new and time-efficient memory-encoding paradigm for functional magnetic resonance imaging (fMRI). This paradigm is optimized for mapping multiple contrasts using a mixed design, using auditory (environmental/vocal) and visual (scene/face) stimuli. We demonstrate that the paradigm evokes robust neuronal activity in typical sensory and memory networks. We were able to detect auditory and visual sensory-specific encoding activities in auditory and visual cortices. Also, we detected stimulus-selective activation in environmental-, voice-, scene-, and face-selective brain regions (parahippocampal place and fusiform face area). A subsequent recognition task allowed the detection of sensory-specific encoding success activity (ESA) in both auditory and visual cortices, as well as sensory-unspecific positive ESA in the hippocampus. Further, sensory-unspecific negative ESA was observed in the precuneus. Among others, the parallel mixed design enabled sustained and transient activity comparison in contrast to rest blocks. Sustained and transient activations showed great overlap in most sensory brain regions, whereas several regions, typically associated with the default-mode network, showed transient rather than sustained deactivation. We also show that the use of a parallel mixed model had relatively little influence on positive or negative ESA. Together, these results demonstrate a feasible, versatile, and brief memory-encoding task, which includes multiple sensory stimuli to guarantee a comprehensive measurement. This task is especially suitable for large-scale clinical or population studies, which aim to test task-evoked sensory-specific and sensory-unspecific memory-encoding performance as well as broad sensory activity across the life span within a very limited time frame.

## Introduction

With neurodegenerative diseases as one of the main challenges in aging populations, the precise, comprehensive, and robust measurement of cognitive functions is of great importance. Functional magnetic resonance imaging (fMRI) is one measurement that helps us to bridge the space between biology and behavioral outcomes. Several large-scale studies have employed fMRI to map brain activity in the general population, including the Rotterdam Study (Hofman et al., 2015), UK Biobank (Miller et al., 2016), and the Rhineland Study (Breteler et al., 2014). These large-scale population studies are usually not designed to answer one specific hypothesis. Rather, they aim to perform an extensive and deep phenotyping that allows addressing multiple questions. As they are mostly prospective studies, they also need to anticipate future questions. Therefore, tasks and paradigms should ideally be as versatile as possible. In the absence of a specific hypothesis, resting-state fMRI is often employed, mostly for practical considerations, as it is rather easy to apply and can also inform about neural dysfunction (Damoiseaux and Huijbers, 2017). Task-evoked fMRI provides complementary information, that is, the brain's response to specific demands (Campbell and Schacter, 2017; Davis et al., 2017), and evokes activity in cortical networks under more restrained conditions (Vanderwal et al., 2015; Huijbers et al., 2017). Therefore, task fMRI is often considered. However, most conventional task paradigms are not easily applied in clinical or large-scale population studies (Pinel et al., 2007) for the following reasons.

First, conventional task paradigms from cognitive neuroscience are typically developed and applied in experimental studies that pose less time constraints than population studies. However, in clinical or large-scale population studies, acquisition time is often more restricted, as the burden to participants, or patients, should be limited and costs add up easily. Additionally, fMRI acquisition time typically competes with anatomical or clinically motivated MRI sequences, including T1, T2, fluid-attenuated inversion recovery (FLAIR), susceptibility-weighted imaging (SWI), perfusion, and diffusion (Jack et al., 2008; Glasser et al., 2013). Thus, to be feasible for clinical or population-based imaging, a task paradigm should be as time-efficient as possible.

Second, conventional fMRI task paradigms have often been developed in homogenous cohorts of young adults. In a large-scale population or clinical studies, the cohort of participants is typically more heterogeneous with respect to age, education, lifestyle, and health factors. This heterogeneity can result in problems when task instructions are tailored to a specific age group (such as young adults). As a consequence, paradigms might show ceiling and/or floor effects for subgroups. Thus, an ideal paradigm should have very simple or no instructions and yet remain informative across the entire cohort.

Finally, conventional task paradigms are typically designed to answer a specific hypothesis, often from the field of cognitive neuroscience. As mentioned above, large-scale, population-based studies mostly aim to employ fMRI to estimate neuronal activity related to multiple research questions or outcomes at the same time. An ideal task paradigm for population-based studies should permit the analysis of multiple contrasts that span a wide range of cognitive functions.

To address these various requirements, we designed a novel task paradigm that we consider especially suited for large-scale studies. It measures predominantly memory encoding, but also perception and attention in both the auditory and visual domains within 10 min of fMRI acquisition time using simple instructions. To our knowledge, memory-encoding paradigms so far presented stimuli of one sensory condition or did face–name associative memory tasks (Sperling, 2007; Barch et al., 2013; Nenert et al., 2014; Sidhu et al., 2015; Hayes et al., 2017) within a similar time frame. We optimized our task to allow mapping of a versatile number of contrasts that are relatively straightforward to interpret. To enable the separation of sensory-specific and sensory-unspecific activities (Wheeler et al., 2000; Daselaar et al., 2010; Langner et al., 2012), we used two sensory modalities, auditory and visual. Twenty-five percent of the total time consisted of passive rest blocks as baseline/rest condition (Gusnard and Raichle, 2001). Each sensory condition contained two distinct sub-conditions to cover a wide range of information on visual and auditory system activations as well as joined activation for sensory-unspecific functions like overall memory. Within the visual condition, we chose to present faces and spatial scenes, motivated by work on face-selective and scene-selective brain regions (Kanwisher et al., 1997; Epstein and Kanwisher, 1998; Gazzaley et al., 2005; Collins and Dickerson, 2019). Further, those stimuli seemed to show differences in age-related reductions in neural dedifferentiation, which makes them interesting for longitudinal studies (Srokova et al., 2020). To select auditory stimuli on a similar level of specificity, we chose voice and environmental stimuli motivated by previous work on voice-selective brain regions (Belin et al., 2000, 2002; Pernet et al., 2015; Agus et al., 2017; Zäske et al., 2017; Aglieri et al., 2018). This decision was further supported by studies showing that similarities as well as differences exist between the regional activation of voice and face perception (Young et al., 2020). Due to the simplicity of the design and to keep the paradigm language free, we did not include language stimuli. A post-fMRI recognition test, with previously seen/heard and novel items, enables the computation of contrasts between subsequently remembered (hit) and forgotten (miss) items (Wagner et al., 1998; Otten and Rugg, 2001; Prince et al., 2009; Collins and Dickerson, 2019). In the following, we will refer to these contrasts as encoding success activity (ESA). We used a parallel mixed block/event design to include a large number of stimuli within a limited time and to enable the already versatile number of contrasts also for the separation of sustained (block) and transient (event) activities (Velanova et al., 2003; Visscher et al., 2003; Petersen and Dubis, 2012). Differentiating both can help to get a more complex understanding of the functional processes underlying a task. Sustained effects give more information about the maintenance of activity throughout a set of stimuli, for example, representing also overall attentional performance or arousal, whereas transient effects are specific for each trial of a task (Visscher et al., 2003).

Thereby, our task allows a large degree of flexibility to analyze the data in multiple ways with regard to other outcomes of interest. This is important in studies spanning years to decades, as research questions and analysis techniques change over time. In this study, we introduce our task paradigm and demonstrate several possible analyses to generate a range of different behavioral and neuronal measures relating to perception and memory encoding. These outcome measures are then available for further analyses in the context of the overall population study.

## Materials and Methods

### Participants

We recruited 60 young adults between the ages of 19 and 30 years (*M* = 24.18, *SD* = 2.90; 36 females), from the University of Bonn community in the context of the pilot studies for the Rhineland Study, a prospective cohort study. The study was carried out in accordance with the recommendations of the International Council for Harmonization (ICH) Good Clinical Practice (GCP) standards (ICH-GCP). We obtained written informed consent from all participants in accordance with the Declaration of Helsinki. No incentives were offered to the participants. The medical ethics committee of the Medical Faculty of the University of Bonn approved the study. All participants had normal or corrected-to-normal vision. Hearing levels were calibrated individually before the experiment, for the sounds to be easily audible above the scanner noise. For one participant, visual retrieval data were not available in the fMRI analysis. ESA contrasts for this participant were therefore analyzed only on the basis of the auditory retrieval information. To detect possible floor effects of the task, we obtained behavioral task data of 21 persons older than 30 years (*M* = 52.71, *SD* = 15.55; age range = 31–77; 12 females) (see Supplementary Material “Behavioral results in older adults”).

### Stimuli

A total of 160 auditory and 160 visual items were presented during the encoding task. Auditory stimuli had durations between 538 and 2,771 ms (*M* = 1,630 ms, *SD* = 488 ms) and consisted of 80 environmental and 80 human vocal sounds. The environmental sounds included a mix of sounds from animals, traffic, tools, and musical instruments, selected from previous auditory experiments (Belin et al., 2000; Daselaar et al., 2010; Huijbers et al., 2011). The vocal sounds consisted of vocal utterances, void of semantic content, such as laughing, crying, or coughing, selected from previous experiments, from the Oxford Vocal (OxVoc) Sounds database (Belin et al., 2000; Parsons et al., 2014) or were recorded for the purpose of this study. The recordings were from various male and female voices. Duration of the auditory stimuli was not equalized, because some are by nature rather short but nevertheless distinct, whereas others need a longer duration to be distinct (e.g., cockcrow/doorbell vs. laughter/wind). To match stimuli for low-level physical properties, we normalized all auditory stimuli to the same amplitude using version 2.0.6 of Audacity® recording and editing software. The visual items consisted of color photographs of 80 faces (size 570 × 360 pixels) and 80 scenes (size 500 × 375 pixels) on a black background. Face stimuli contained faces from individuals with various ethnicities, between 18 and 90 years of age, with an equal number of male and female faces selected from previous experiments (Sperling et al., 2003; Minear and Park, 2004; Huijbers et al., 2015). Scenes were pictures from nature or urban outdoor environments selected from Huijbers et al. (2009). Colors from the original scenic images were slightly de-saturated to match the color contrast in the facial images.

From all available stimuli, we selected the final set of 160 stimuli with the aim to reach a hit-rate of ~50% (for an explanation, see section Behavioral Analysis) and a false alarm (FA) rate as low as possible.

Auditory stimuli were presented via S14 Insert Earphones (Sensimetrics, Malden, USA). Visual stimuli were presented on a screen located at the head of the magnet bore and seen via a mirror mounted on the head coil. All stimuli were presented using PsychoPy software v1.82 (Peirce, 2007), running on a Windows PC.

### MRI Acquisition

fMRI data were acquired with a 3-Tesla Siemens MAGNETOM Prisma system (Siemens Medical Systems, Erlangen, Germany). The scanner was equipped with a 64-channel phased-array head/neck coil. We used inflatable air pads to restrict head movement, and participants were instructed to lie still for the duration of the scan. For the applicability in large-scale testing, we decided on a standard fMRI scanning protocol: we acquired two task fMRI sessions of 140 volumes using echo-planar imaging (EPI), including four dummies. Each volume consisted of 32 axial slices of 3-mm thickness with a 0.75-mm gap. The repetition time (TR) was 2,000 ms, echo time (TE) was 30 ms, flip angle was 84°, readout bandwidth was 2,300 Hz/pixel, the slice orientation was anterior commissure–posterior commissure (AC-PC), and field of view (FOV) was 192 × 192 mm, resulting in an effective voxel size of 3.0 × 3.0 × 3.75 mm.

### Task Design and Implementation

**Figure 1:**
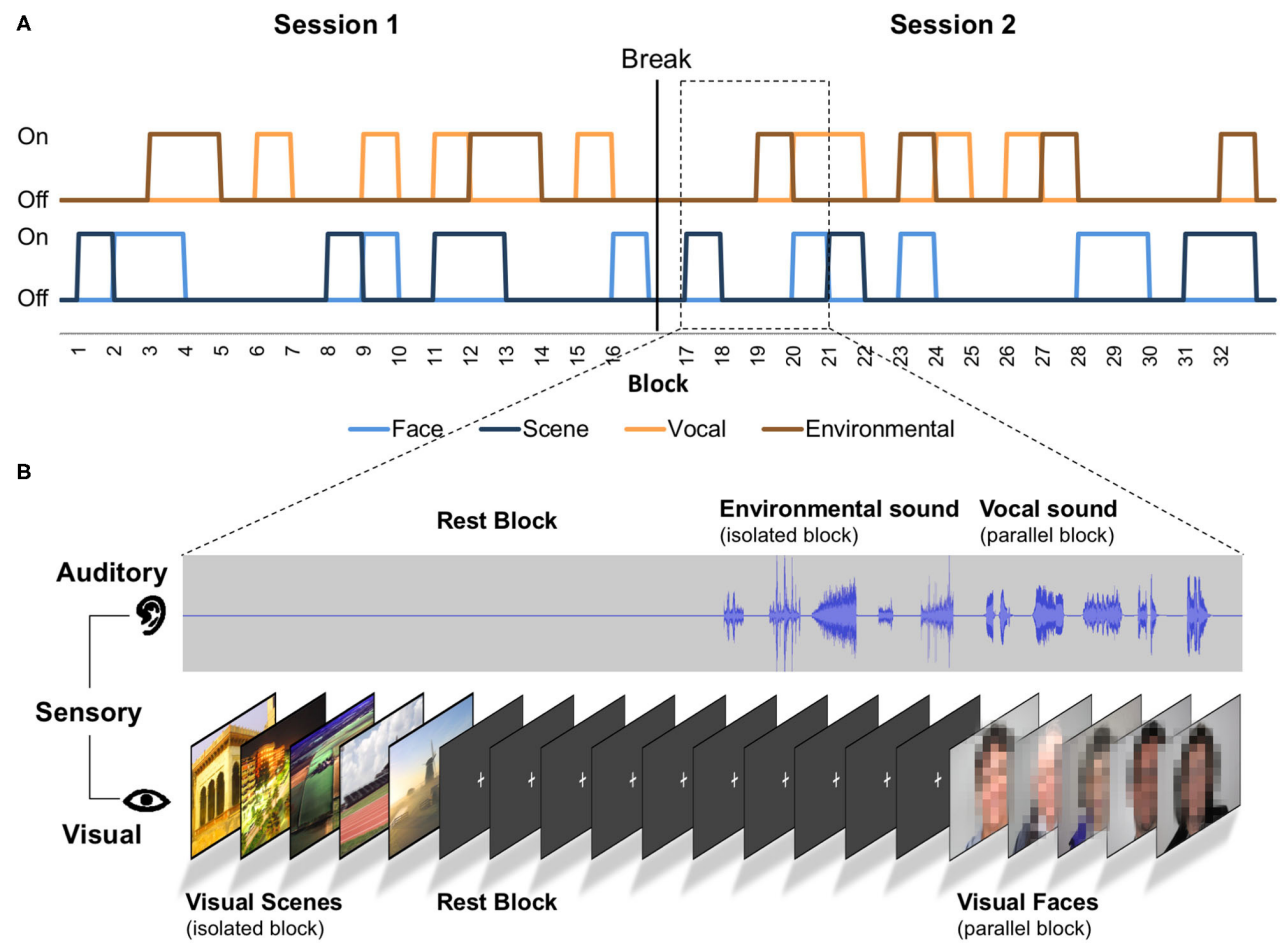
Scheme of the task paradigm. **(A)** The order of presented stimuli and sensory conditions. “On” represents presentation of the stimuli, and “Off” represents no presentation of the stimuli. Each block contains five stimuli of one category and lasted 16 s. In parallel conditions, five auditory and five visual stimuli are presented in one block. The auditory events consisted of environmental or vocal stimuli (see auditory timeline). The visual events consisted of face or scene stimuli (see visual timeline; faces were not pixelated in the original task). **(B)** Four exemplary blocks in detail.

The task was designed as a mixed model (Visscher et al., 2003) and included 180 events (trials), grouped into 32 blocks (Figure 1). Out of these 32 blocks, eight were rest blocks (fixation), eight isolated auditory blocks, eight isolated visual blocks, and eight parallel auditory/visual blocks. Sixteen blocks contained auditory stimuli, half isolated auditory and half parallel with visual images. Of these 16 blocks, eight blocks contained environmental sounds (four isolated and four parallel), and eight blocks contained vocal sounds (four isolated and four parallel). Similarly, for the visual blocks, half were presented in isolation and half in parallel with sounds (scene and face images equally distributed). In each block, five items—sounds, images, or both—were presented for a total of 16 s per block (Figure 1). Within the auditory blocks, the inter-trial interval between items was 200–2,700 ms. Within the visual stimulus blocks, the inter-trial interval between items was 200–2,200 ms. The difference in the inter-trial intervals between auditory and visual blocks is due to the variable duration of sounds. Each image was presented for exactly 2,000 ms. Inter-trial intervals as well as the order of blocks and the order of stimuli within the blocks were once randomly assigned and remained the same for all participants. A white fixation cross on black background was shown during the rest blocks, the inter-trial intervals, the isolated auditory blocks, and the initial and final 8 s of each run. By design, we tried to ensure that the different blocks and items would result in separate, uncorrelated regressors (see section Parallel Mixed Model Analysis). As we cannot predetermine which items will be remembered or forgotten, we also evaluated the collinearity of the regressors after data collection (see section Parallel Mixed Model Analysis).

The task presentation was distributed over two sessions (containing the same number of blocks for each stimulus and presentation condition) of 4:54 min each separated by a short question about the participants' well-being. At the beginning of these sessions, participants were given written instructions via the screen to pay attention to the sounds and images (“please watch and listen carefully”). No motor responses were required in our task, which had several advantages. Apart from keeping the task simple, movement artifacts during scanning were minimized. Also, the lack of motor activity facilitates the interpretation of sensory and memory-related fMRI data across the life span (Yarkoni et al., 2009; Viswanathan et al., 2020).

Before the fMRI sessions, vision and hearing abilities were corrected to normal, by using MRI-compatible glasses and a volume adjustment during the initial scout scan, respectively. Following the scout scan, participants did a very short training session of eight visual and eight auditory items including encoding and retrieval, to get acquainted with the task procedures and to ensure they understood the instructions. Our following encoding task was therefore explicit. After completing the two memory-encoding sessions, participants' memory retrieval was tested by two separate subsequent memory tests. Recognition of auditory stimuli was tested first, followed by a visual recognition test. Across the auditory and visual recognition tests, 160 previously encoded (old) and 160 novel (new) items were presented (80 environmental sounds/80 vocal sounds/80 face images/80 scene images). The participants responded with two buttons (“Yes” and “No”) to a forced-choice question (in German): “Did you hear/see this item previously?” (“Haben Sie das Geräusch bereits gehört?” or “Haben Sie das Bild bereits gesehen?”). The recognition tests were self-paced, and items were presented in blocks. In each block, five old items (previously encoded) and five new items were presented in a random order. In each block, items were of the same type. Across the blocks, the presentation order of the encoding intervals was maintained to ensure an approximately equal time distance between encoding and retrieval. Both recognition tests were done inside the MRI bore immediately after the encoding runs. The auditory recognition test was done during a diffusion MRI scan, and the visual recognition test was performed during an anatomical T1-MPRAGE scan. Diffusion and anatomical MRI data are not included in this manuscript, yet some of that data have been examined in relation to head motion (Huijbers et al., 2017). The fMRI and behavioral data in this paper have not been published previously. All task scripts are uploaded under https://www.rheinland-studie.de/data-code/boenniger2020.

### Behavioral Analysis

Behavioral analyses were implemented in R v3.3.2 (http://www.r-project.org/). To quantify memory performance, we examined the percentage of correct responses for previously presented items (labeled as hit-rate) and the percentage of incorrect responses for new items (labeled as FA-rate). We also calculated the discriminability index d-prime (*d*′), by taking the z-standardized hit-rate minus the z-standardized FA-rate. Additionally, we calculated the response bias (*c*) by taking the sum of the z-standardized hit- and FA-rates multiplied by −0.5. Differences between hit- and FA-rates were calculated using paired and two-sided *t*-tests. A one-sample *t*-test was used to examine the response bias. To assess the main effects and the possible interaction between sensory modality (auditory/visual) and presentation condition (isolated/parallel) on memory performance, we used an ANOVA. Correlation analyses described in the supplements employ Pearson's method, unless otherwise indicated. Reliability analysis was done by splitting up the task into its two sessions (for details, see Supplementary Material “Analysis of reliability”) and calculating the intraclass correlation coefficients (ICC) (Shrout and Fleiss, 1979; McGraw and Wong, 1996) with a two-way model using single units for each participant, estimating the consistency between the two sessions.

### Functional MRI Preprocessing

fMRI data were preprocessed using MATLAB (MathWorks, Natick, MA, USA), the Statistical Parametric Mapping Toolbox (SPM8, UCL, London, UK), and GLM Flex (MGH, http://mrtools.mgh.harvard.edu/index.php/GLM_Flex, MA, USA). First, we dropped the four dummy volumes. Second, we realigned the time series to the first volume. Third, we normalized the data to a standard EPI template in Montreal Neurological Institute (MNI) 152 space. Fourth, we smoothed the data with a full-width-half-maximum (FWHM) kernel of 8 mm. For assessing the reliability, we split the task into two sessions (for details see Supplementary Material “Analysis of reliability”) calculated on the basis of the slice time-corrected and normalized data. We calculated ICC values before smoothing the data, using a publicly available online script for MATLAB by C. Pernet (https://github.com/CPernet/spmrt/blob/master/spmrt_fMRI_ICC.m, downloaded 8th February 2018). After calculating the ICCs on the voxel level, we smoothed the group-level ICC maps with a FWHM kernel of 8 mm for visualization purposes, as this makes it easier to appreciate the spatial overlap between the contrast and the ICC map.

### Parallel Mixed Model Analysis

The subject-level analyses were conducted in SPM8. For the main analyses, the SPM regressors were modeled according to the parallel mixed block/event design (Visscher et al., 2003). In the Supplementary Material, we also added a model comparison where we modeled the task data according to a block-only design and an event-only design using the respective regressors separately (see Supplementary Material “Model comparison between mixed, block- and event-only modeling”).

**Figure 2:**
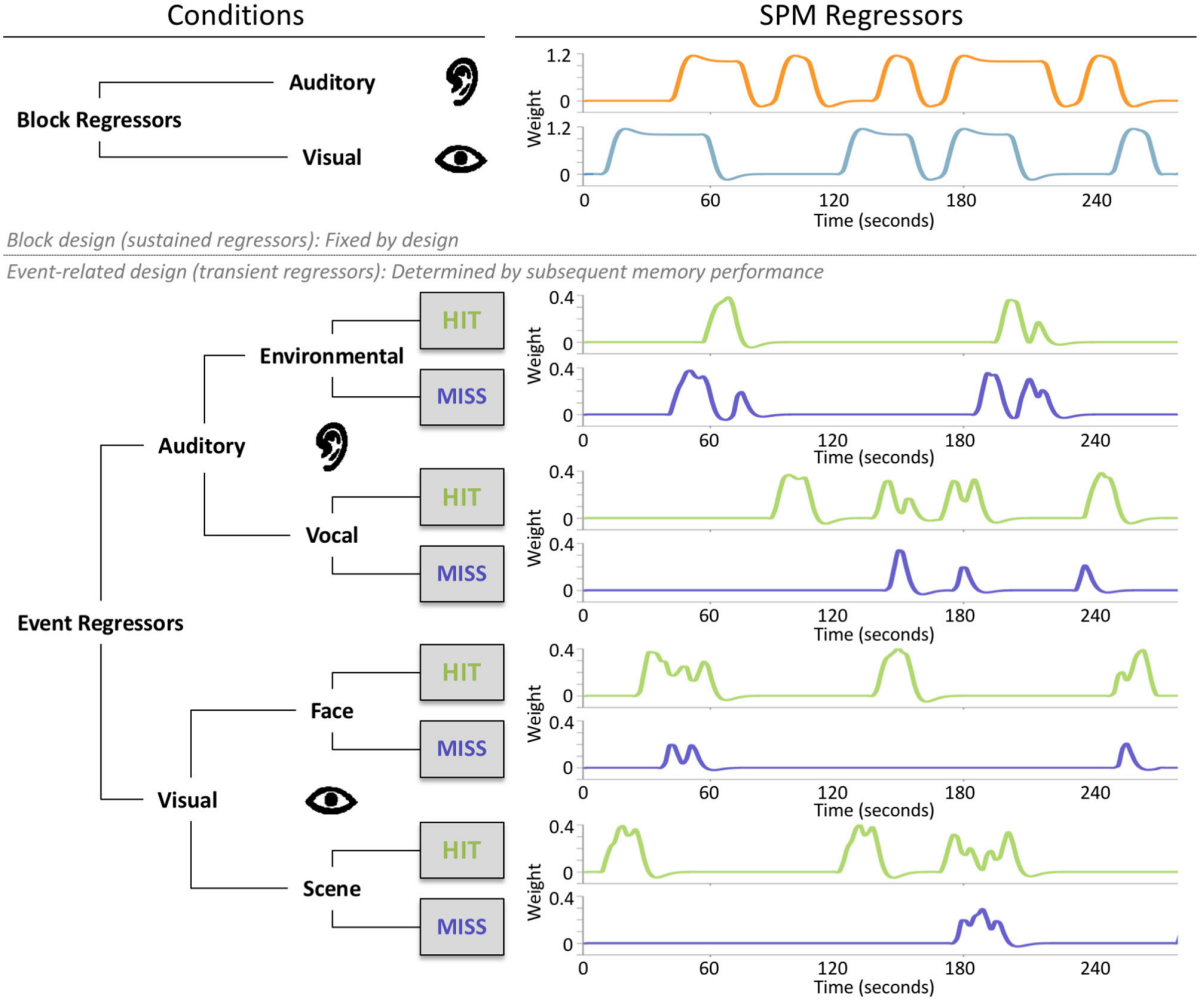
The parallel mixed design consisted of two fixed block regressors, one for the auditory and one for the visual blocks, and eight subject-specific event regressors. These event-related regressors were determined by the combination of stimulus types, auditory (environmental/vocal) or visual (face/scene) and subsequent memory performance (hits/misses). Event regressors represent the SPM regressors of a single exemplary subject.

In the mixed design, we included two block regressors: one for the auditory blocks and one for the visual blocks (Figure 2). The block onsets were convolved with the canonical hemodynamic response function using the durations. Passive rest blocks (fixation) were not modeled explicitly. The block regressors were solely determined by the task design and therefore fixed across subjects. In addition, we also added eight unique event regressors, which were subject specific, as they were determined by the combination of the task design (stimulus type: environmental sounds/vocal sounds/face images/scene images) and the participants' performance on the subsequent memory tasks (memory performance: hit/miss) (Figure 2). To avoid collinearity (Andrade et al., 1999), we included the parallel (auditory and visual) presentation condition in an equal amount to the isolated (auditory or visual) and rest (fixation) blocks to the task. In addition, we modeled blocks for both sensory conditions (auditory/visual), whereas events were modeled for each stimulus condition (environmental sounds/vocal sounds/scene images/face images) separately. To prevent correlations between regressors due to participants who remembered or forgot too many items (events) presented within one block, we aimed for a conservative response bias (see section Behavioral Results). This also ensured roughly equal hits/misses, so hits or misses did not dominate single blocks. After data collection, before the analysis, we checked the hemodynamic regressors for collinearity using a correlation analysis. All regressors have shown to be largely independent with *r* = 0.3 within sensory conditions and r around zero between sensory conditions. On average, the event-related regressors were modeled based on 20.65 environmental hits (*SD* = 5.87), 19.25 environmental misses (*SD* = 5.77), 24.63 vocal hits (*SD* = 6.35), 15.30 vocal misses (*SD* = 6.30), 26.42 scene hits (*SD* = 6.16), 13.58 scene misses (*SD* = 6.16), 23.73 face hits (*SD* = 6.64), and 16.27 face misses (*SD* = 6.64). The event onsets were convolved with the canonical hemodynamic response function using no duration. Further, the subject-level models also included regressors for motion parameters, bad-volume regressors, and a high-pass filter (1/128 Hz). The bad volumes were defined by the amount of absolute movement in relation to the previous scans, using a threshold >0.75 mm or 1.5° in one or more directions.

We defined 10 contrasts. First, the block-based contrast was performed between auditory and visual blocks (c1) to assess activity caused by the different sensory conditions. Second, two event-related contrasts based on stimulus type was performed to assess within the sensory conditions differences between stimuli types: (c2) environmental sounds vs. vocal sounds masked by auditory activity greater than visual activity and (c3) face images vs. scene images masked by visual activity greater than auditory activity. Third, we defined ESA based on the events during isolated blocks: (c4) all (visual and auditory) hits vs. all misses (sensory-unspecific ESA), (c5) auditory hits vs. auditory misses (auditory ESA), and (c6) visual hits vs. visual misses (visual ESA). We included ESA only for each sensory type (auditory and visual stimuli), as we considered the type-specific ESA maps (environmental sounds, vocal sounds, scene images, and face images) to be too detailed and to have not enough trials. Fourth, we defined contrasts relative to the rest condition (fixation), to examine differences and similarities between sustained and transient activities: (c7) auditory blocks vs. rest, (c8) isolated auditory events vs. rest, (c9) visual blocks vs. rest, and (c10) isolated visual events vs. rest.

For all group maps, we used a global threshold of *p* < 0.05 [false discovery rate (FDR) corrected] with a minimum cluster size of five voxels (no cluster-size correction). The same threshold was used to define the masks for the conjunction analyses (c2/c3). Note that the remaining activities within the mask also had to survive the global threshold (*p* < 0.05, FDR corrected). Statistical group maps were projected to the cortical surface using FreeSurfer (v5.1) via a standard MNI to the FreeSurfer average template transformation or were resliced to 2.0 × 2.0 × 2.0 mm voxels and overlaid on the standard SPM8 individual T1-weighted volume.

## Results

### Behavioral Results

The subsequent memory performance is listed in Table 1. For each sensory and stimulus condition, the hit-rate was significantly greater than the FA-rate. These differences between the hit- and FA-rates indicate that participants were able to successfully encode items in each category. The duration of subsequent memory test was between 10 and 19 min. The auditory retrieval took on average 7.32 min (*SD* = 0.86, range 6.40–11.16) and visual retrieval 3.86 min (*SD* = 0.77, range 3.03–8.18). Within the auditory blocks, the inter-trial intervals for hits and misses were on average 1,556 ms (range 194–2,713 ms) and 1,509 ms (range 194–2,713 ms), respectively. Within visual blocks, the inter-trial intervals for hits and misses were on average 1,136 (range 200–2,200 ms) and 1,171 ms (range 200–2,200 ms), respectively.

**Table 1:** Memory performance.

	**Hit-rate**		**FA-rate**		**Hit-rate vs. FA-rate**			***d***^****′****^				
	***M***	***SD***	***M***	***SD***	***t***	**df**	***p***	***M***	***SD***	***t***	**df**	***p***
Auditory	0.57	0.14	0.23	0.13	17.13	59	<0.001	0.99	0.46			
Visual	0.63	0.14	0.15	0.12	21.81	59	<0.001	1.48	0.58			
Auditory vs. Visual										6.09	59	<0.001
Environmental	0.52	0.15	0.19	0.11	15.73	59	<0.001	1.02	0.54			
Vocal	0.62	0.16	0.27	0.16	15.80	59	<0.001	1.02	0.50			
Environmental vs. Vocal										0.00	59	1.000
Face	0.59	0.17	0.19	0.15	17.35	59	<0.001	1.21	0.61			
Scene	0.66	0.15	0.11	0.11	22.88	59	<0.001	1.87	0.75			
Face vs. Scene										9.47	59	<0.001

*Mean (M) and standard deviation (SD) for hit-rate, false alarm (FA)-rate, and d-prime (d′) (N = 60). Paired t-tests are used to depict differences between hit- and FA-rates and between d′ of sensory and stimulus conditions. df indicates the degrees of freedom, t indicates the t-value, and p indicates the p-value representing the significance level*.

Across all conditions, we found a *d*′ of 1.19 (*SD* = 0.40) and a *c* of 0.34 (*SD* = 0.27) [auditory: *c* = 0.32 (*SD* = 0.33); visual: *c* = 0.39 (*SD* = 0.33)]. The response bias indicated that participants were relatively conservative [*t*_(59)_ = 9.59, *p* < 0.001] and thus more likely to rate items as “new.” Paired *t*-tests indicated that memory performance was better for visual items compared with auditory items and for scene images better than for face images, but there was no difference in memory performance between environmental and vocal auditory stimuli (Table 1). Similar results, with slightly lower *d*′ but a comparable response bias, have been observed for the small sample of older participants (see Supplementary Material “Behavioral results in older adults”).

In our paradigm, stimuli were presented either in isolation or in parallel with stimuli of the other sensory modality. We computed separate *d*′ values for each of the presentation conditions (parallel/isolated) and sensory condition (auditory/visual). *d*′ values for subsequent memory of auditory stimuli were *M*_*isolated*_ = 1.03 (*SD*_*isolated*_ = 0.51) and *M*_*parallel*_ = 0.95 (*SD*_*parallel*_ = 0.45) and for visual stimuli *d*′ values of *M*_*isolated*_ = 1.69 (*SD*_*isolated*_ = 0.70) and *M*_*parallel*_ = 1.31 (*SD*_*parallel*_ = 0.60). Results of the ANOVA supported a better subsequent memory performance for visual than for auditory stimuli independent of the presentation condition (isolated/parallel) [*F*_(1, 59)_ = 37.29, *p* < 0.001]. Also the presentation condition showed a main effect indicating a better subsequent memory performance for items presented in isolation independent of the sensory modality [*F*_(1, 59)_ = 40.60, *p* < 0.001]. Further, we found an interaction effect between sensory modality and presentation condition [*F*_(1, 59)_ = 25.62, *p* < 0.001], which suggested that the parallel presentation of auditory and visual items was more detrimental to learning of visual information than of auditory information.

### Sensory-Specific Activity

**Figure 3:**
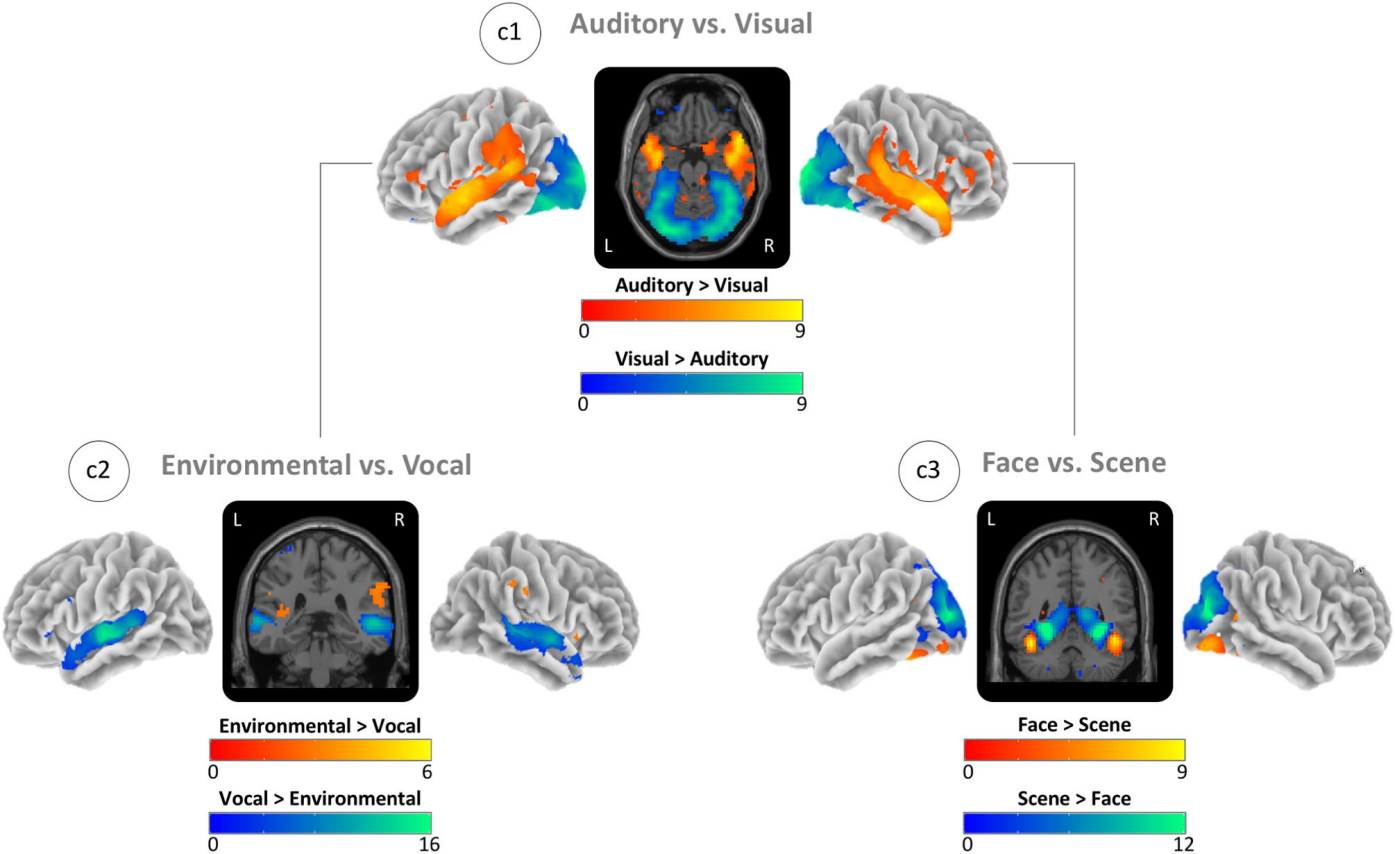
Task-based activity contrasts between (Auditory vs. Visual) and within (Environmental vs. Vocal and Face vs. Scene) sensory conditions. (c1) Block-based contrast between auditory and visual stimulus blocks. (c2) Event-related contrast between environmental and vocal sounds masked by auditory greater visual activity (see c1). (c3) Event-related contrast between face and scene images masked by visual greater auditory activity (see c1). Brain activity is shown at a threshold of *p* < 0.05 [false discovery rate (FDR) corrected], and the color intensity shows the *t*-value. Contrast maps are uploaded under https://neurovault.org/collections/IABCOPVN/.

For the auditory blocks, we found the global maxima in the right auditory cortex and for the visual blocks in the left visual cortex (Figure 3; c1). For the environmental sounds, the maxima were in the right temporoparietal junction, and for the vocal sounds, in the right superior temporal gyrus (Figure 3; c2). For scenes, we found maxima in the left parahippocampal gyrus, and for faces, in the left fusiform gyrus (Figure 3; c3). See sensory-specific activity in Table 2 for the MNI coordinates and values of the global maxima (activation) and minima (deactivations). Supplementary Table A.2 in the supplement provides cluster specific peaks for all contrasts.

**Table 2:** Maximally activated and deactivated brain regions for each contrast.

**Contrast**		**Region**	**MNI_**(x, y, z)**_**	***t*-value**	**BA**
**Sensory-specific activity**					
c1	Auditory > Visual	Auditory cortex	54, −1, −13	9.90	22/41
	Visual > Auditory	Visual cortex	−3, −91, −4	13.13	17/18
c2	Environmental > Vocal	Temporoparietal junction	57, −28, 32	3.78	39/40
	Vocal > Environmental	Superior temporal gyrus	63, −1, −10	11.65	22
c3	Face > Scene	Fusiform gyrus	−42, −49, −22	8.17	37
	Scene > Face	Parahippocampal gyrus	−27, −49, −7	24.08	19/36
**Encoding success activity (ESA) for isolated blocks**					
c4	Positive ESA	Hippocampus	21, −7, −25	6.09	36/54
	Negative ESA	Precuneus	9, −70, 44	−6.37	7
c5	Positive auditory ESA	Auditory cortex	−60, −13, −4	5.72	22
	Negative auditory ESA	Precuneus	21, −55, 23	−5.06	23
c6	Positive visual ESA	Visual cortex	27, −91, −4	5.31	18
	Negative visual ESA	Precuneus	12, −67, 32	−5.53	7/31
**Sustained (blocks)**					
c7	Auditory > rest (activation)	Superior temporal lobe	54, 2, −13	9.71	22
	Auditory < rest (deactivation)	Visual cortex	18, −100, 8	5.39	18
c9	Visual > rest activation)	Primary visual cortex	−6, −88, −1	14.81	17
	Visual < rest (deactivation)	Temporoparietal junction	−63, −25, 26	6.01	40
**Transient (events)**					
c8	Auditory > rest (activation)	Primary auditory cortex	−42, −28, 8	7.89	41
	Auditory < rest (deactivation)	Putamen	21, 8, −13	7.10	49/52
c10	Visual > rest (activation)	Fusiform gyrus	30, −55, −13	8.79	37
	Visual < rest (deactivation)	Inferior temporal gyrus	−30, −49, 5	7.15	19/37

*Contrasts are as follows: c1, block-based contrast between auditory vs. visual stimulus blocks; c2, event-related contrast between environmental vs. vocal sounds; c3, event-related contrast between face vs. scene images; c4, ESA for all (visual and auditory) hits vs. all misses; c5, ESA for auditory hits vs. auditory misses; c6, ESA for visual hits vs. visual misses; c7, auditory vs. rest blocks; c9, visual vs. rest blocks; c8, auditory events vs. rest; and c10, visual events vs. rest. All brain regions are described with Montreal Neurological Institute (MNI) coordinates [MNI_(x, y, z)_], t-values of the beta coefficients, and the related Brodmann area (BA). Contrast maps are uploaded under https://neurovault.org/collections/IABCOPVN/. Positive ESA: hits > misses contrast; negative ESA: misses > hits contrast*.

### Encoding Success Activity

**Figure 4:**
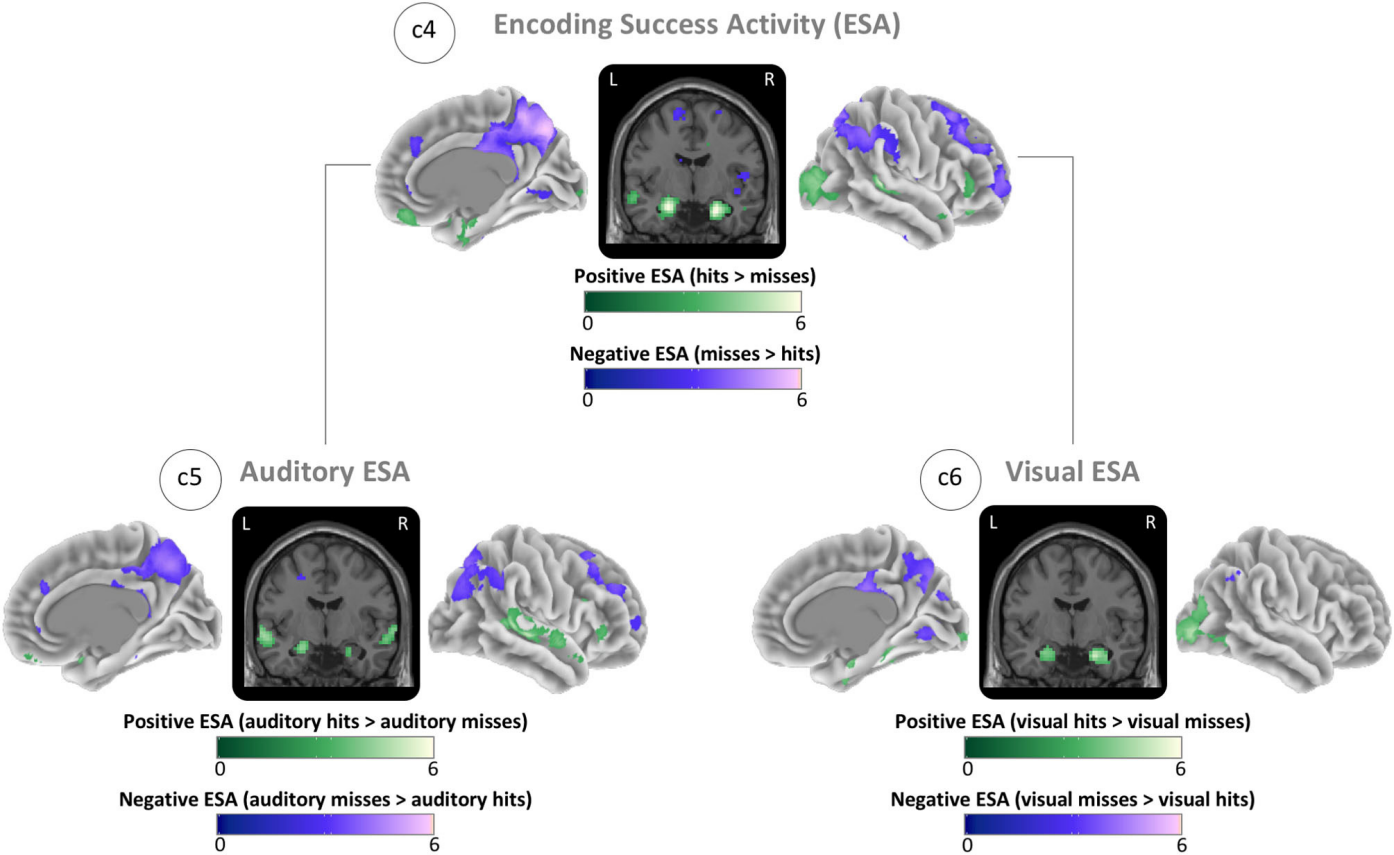
Brain maps of sensory-unspecific encoding success activity (ESA), assessed through the contrasts between activity of subsequently remembered (hit) and subsequently forgotten (miss) stimuli of the isolated encoding condition. (c4) ESA across all conditions (auditory and visual). (c5) ESA of auditory stimuli. (c6) ESA of visual stimuli. Brain activity is shown at a threshold of *p* < 0.05 [false discovery rate (FDR) corrected], and the color intensity shows the *t*-value. Contrast maps are uploaded under https://neurovault.org/collections/IABCOPVN/.

The ESA contrast showed the greatest positive ESA (hits > misses) (global maximum) in the right hippocampus and the greatest negative ESA (misses > hits) (global minimum) in the right precuneus (Figure 4; c4). For auditory items, we found the maximum positive ESA in left auditory cortex and the maximum negative ESA in the right precuneus (Figure 4; c5). For visual items, we found the maximum positive ESA in the right visual cortex and the maximum negative ESA in the right precuneus (Figure 4; c6). See ESA in Table 2 for the MNI coordinates and values and Supplementary Table A.2 for all cluster specific peaks.

Together, these maps demonstrate that positive ESA in the auditory and visual cortices is sensory-specific while the positive ESA in hippocampus and the negative ESA in the precuneus are sensory-unspecific.

### Sustained and Transient Activations

**Figure 5:**
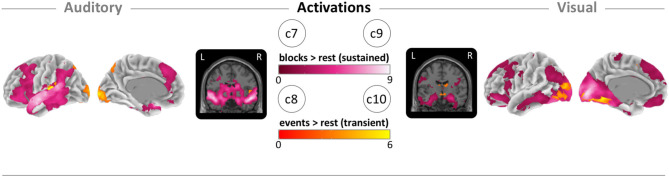
Brain maps of block-related (sustained) and event-related (transient) activations. (c7) Auditory block vs. rest activation (pink). (c8) Auditory event vs. rest activation (orange). (c9) Visual block vs. rest activation (pink). (c10) Visual block vs. rest activation (orange). Brain activity is shown at a threshold of *p* < 0.05 [false discovery rate (FDR) corrected], and the color intensity shows the *t*-value. Contrast maps are uploaded under https://neurovault.org/collections/IABCOPVN/.

To clarify the patterns of sustained and transient activations, we mapped the block- and event-related activity vs. the rest condition for each sensory condition. For sustained (block-based) auditory activity, we found the global maxima in the right superior temporal lobe (Figure 5; c7). For the transient (event-based) auditory activity, we found the maxima in the left primary auditory cortex (Figure 5; c8). For sustained visual activity, we found the maxima in the primary visual cortex (Figure 5; c9). Finally, for transient visual activity, we found the maxima in the right fusiform gyrus (Figure 5; c10). For MNI coordinates and values, see sustained and transient section in Table 2; and for cluster specific peak activation, see Supplementary Table A.2. We also examined the local minima (deactivations) of the same contrasts for which the results and images can be found in the Supplementary Material “Sustained and transient deactivation”.

In comparison with the results from the block-only or event-only models, mixed models show slightly different levels of activity in the regions of interest. The directionality and the appearance of the main effects stayed the same (see Supplementary Material “Model comparison between mixed, block- and event-only modeling”).

### Reliability Analysis

ICCs for all calculated behavioral outcomes in total (hit-rate, FA-rate, and d-prime) and separated for sensory and stimulus conditions ranged between 0.400 and 0.812, with the highest ICC in FA-rates and the lowest in stimulus-specific *d*′ and hit-rates. Overall, *d*′ showed an ICC of 0.675. Sensory-specific *d*′ showed ICCs of 0.622 for auditory and 0.649 for visual stimuli. More detailed results are described in Supplementary Material “Analysis of reliability”.

Smoothened voxel-wise ICC analysis for the fMRI data revealed for all contrasts stronger reliability for regions that showed also high activation. In c1, the visual and auditory cortices showed the highest reliability with a global peak of ICC = 0.663 in the left middle occipital gyrus (MNI_(x, y, z)_: −12, −100, −1). In c2, ICCs are lower but still showed a global peak of ICC = 0.206 in the right auditory cortex (middle temporal gyrus, MNI_(x, y, z)_: 57, −37, 5). In c3, ICC values had a similar range as in c1 with a global peak of ICC = 0.608 in the right fusiform gyrus (MNI_(x, y, z)_: 33, −49, −10). C4 showed a maximum ICC of 0.306 in the right fusiform gyrus (MNI_(x, y, z)_: 27, −82, −13). Other local peaks of ICC in c4 are found, for example, in the right hippocampus (ICC = 0.132, MNI_(x, y, z)_: 15, −4, −16) and the left parahippocampal region (ICC = 0.230, MNI_(x, y, z)_: −15, −4, −19). More detailed results are shown in the Supplementary Material “Reliability of sensory-specific and encoding success activity”.

## Discussion

We demonstrate the feasibility of a parallel mixed design as an efficient strategy for acquisition of rich fMRI data in limited time. The acquired data can give information about sensory-specific brain activation as well as sensory-specific and sensory-unspecific memory performance (taking the behavioral retrieval task data into account) using the key contrasts c1 to c6. The additional contrasts c7 to c10 show that also information on the difference of sustained (block-based) and transient (event-based) models and resulting activation can be obtained.

### Behavior

In the retrieval task, participants showed a hit-rate close to 50%, which is optimal for ESA modeling, as it ensures a balanced number of observations on each side of the contrast (hits vs. misses). Furthermore, FA-rates were very low, which was reflected in a conservative response bias and resulted in *d*′ values far above chance for each stimulus category (environmental and vocal sound, and scene and face images). The *d*′ values far above chance suggested that the ESA contrast is driven by memory encoding and not guessing. Although the hit-rates were all close to 50%, we see slight differences between stimulus conditions (Table 1). Therefore, we cannot rule out completely that some stimulus conditions influenced the weighting and the ESA contrasts and caused small differences between the conditions. Due to the low number of stimuli and the between-subject variance, we did not have sufficient observations for reliable ESA in each separate stimulus category in this sample. If this task is applied in larger studies, there would be also interesting contrasts to examine. For now, we focused on examining auditory ESA, visual ESA, and (overall) ESA.

Already during task construction, we found visual memory performance to be superior for visual scenes compared with visual faces or sounds. This was despite our initial (design) objective to achieve balanced memory scores for each stimulus type. Given this objective, auditory retrieval was tested before visual retrieval, so the time delay between encoding and retrieval for visual stimuli was longer than for auditory stimuli. We enriched the auditory experience by using various speakers for the vocal sounds and the environmental sounds by presenting a large range of stimuli from animals to vehicles. Finally, we degraded the visual scenes slightly by desaturating the originally bright colors of the images (Huijbers et al., 2009). Nevertheless, memory performance for the visual items remained superior, especially for the scenes (Table 1). This finding replicated behavioral work that indicated better memory performance for visual scenes than for any kind of auditory stimuli (Cohen et al., 2009). In general, visual stimuli are more often remembered and with more detail in comparison with auditory memory, if recalled immediately (Thelen et al., 2015; Gloede and Gregg, 2019). However, after a time delay, auditory memories are more stable than visual memory (Gloede and Gregg, 2019). This may imply that it may be more difficult to encode auditory than visual stimuli. In addition, auditory stimuli were presented above the rhythmic scanner noise, whereas visual stimuli were presented in a dark and visually “quiet” environment. Although we took care to select stimuli that were distinctly different from the scanning sounds with regard to spectral and temporal structure, we must consider the possibility that some acoustic masking occurred. The noisy scanning environment creates additional challenges for the auditory system on several levels of processing, in particular for the detection of signal in noise as well as asking for greater attentional demands and efforts. With regard to applying this paradigm in studies with a wider age range, it must be considered that complex listening skills (such as processing speech in noise) decline with age even when controlling for overall hearing thresholds. Also, considering the blood oxygenation level-dependent (BOLD) effect, it is likely that the continuous scanner noise resulted in continuously high activity in the auditory areas, rendering it more difficult to detect more subtle effects of condition on top of this saturation effect (Tomasi et al., 2005). However, we decided against presenting auditory stimuli in quiet(er) pauses (between volumes), as such a sparse sampling paradigm would have significantly extended the scanning time. In future developments of such paradigms, and especially in studies including older adults, novel methodological approaches such as interleaved silent steady state or special scanning sequences that minimize acoustic impact could be considered [see methodological review by Peelle (2014)].

Looking into differences between the two presentation conditions (isolation and parallel presentation), we also observed differences in memory performance. Isolated presentation resulted in better memory performance for both visual and auditory items. This is consistent with the model that memory encoding is limited by a working memory capacity (Baddeley, 2003) and that it is impaired if semantically incongruent information is presented in parallel (Thelen et al., 2015). One interpretation is that divided attention between auditory and visual information is detrimental to encoding. Note that we tested the auditory and visual retrieval separately, and the information of the two parallel presented stimulus classes was not congruent. There is a large body of evidence that multisensory encoding of congruent information is beneficial for memory performance [Shams and Seitz, 2008; Thelen et al., 2015), for review, see (Quak et al., 2015)]. Therefore, depending on the scientific aim, one could adapt our paradigm and match voices with faces and scenes with environmental sounds. By doing so, memory performance is likely to improve at the expense of either the factorial design or a longer acquisition period.

Interestingly, the relative difference between isolated and parallel encoding was not the same for auditory and visual stimuli. We found that visual memory performance declined more under parallel conditions, while auditory encoding was less hindered. This has also been found in a working memory study on isolated and parallel retention of auditory (vocal) and visual (abstract objects) information (Saults and Cowan, 2007). Together with the finding from Gloede and Gregg (Gloede and Gregg, 2019) that visual memory is more hindered by a delayed recall than auditory memory, this suggests that auditory encoding might be more difficult but relatively robust. One explanation for the robustness of auditory encoding over the presentation conditions might also be related to the noisy scanner environment, creating continuously higher demands on auditory processing during both presentation conditions, as discussed above. This might have reduced the size of the effect of additional between-modality parallel processing for the auditory stimuli. A second explanation could be that learning auditory stimuli in similar detail as visual memory is more difficult and takes more attentional effort (Gloede and Gregg, 2019). Therefore, parallel conditions that demand more attention influence auditory information less than visual memory.

As discussed above, *d*′ values showed that the participant's memory scores were far above chance. Within the young adults, we did not find ceiling effects, and we did not find floor effects in the older adults (see Supplementary Material “Association between age and memory performance”). Together, this makes the task suitable for a life span study. We also assume that our task can be performed by participants with cognitive impairment and dementia. Sperling et al. (2003) showed that mild Alzheimer's disease patients were able to do a face–name association task in which participants had to remember which name was associated with which face. In comparison, we had similar to even less instructions in our encoding task, and our retrieval task was easier, as we probed recognition memory, via old/new judgment, and not associative memory with previously seen lures. Further, for the recognition task, the questions and the answering options were shown on the screen for each trial. In conclusion, as long as participants are willing to be scanned for at least 10 min, the task should be applicable for people across all age ranges as well as people affected by neurodegenerative diseases.

### Sensory-Specific Activity

Mapping of perceptive auditory and visual brain activity (Figure 3: c1) showed quite consistent results with previous work with activity for auditory conditions in environmental and vocal selective brain regions (Belin et al., 2000; Pernet et al., 2015; Agus et al., 2017; Young et al., 2020) and with activity for visual conditions in face- and scene-selective brain regions, i.e., the fusiform face and the parahippocampal place area (Kanwisher et al., 1997; Epstein and Kanwisher, 1998; Gazzaley et al., 2005; Collins and Olson, 2014; Young et al., 2020). Response strength differences for vocal vs. environmental sound stimuli are also consistent with previous work (Belin et al., 2000, 2002; Mostafa, 2012). This imbalance could reflect either the properties of the auditory stimuli (spectral frequencies and temporal structure) or the organization of the auditory system. Although we found slightly stronger responses to scene stimuli, the contrast between face and scene stimuli was more balanced. These more similar levels of activity might also reflect either some property of the visual stimuli (i.e., similar discriminability) or the organization of the visual system.

From a design perspective, the relative imbalance in evoked auditory activity is suboptimal. We mostly used stimuli from previous experiments to replicate known activity patterns by using the parallel mixed design (Belin et al., 2000; Sperling et al., 2003; Huijbers et al., 2009). The application of two different stimulus conditions for visual and auditory senses allowed a detailed examination of the sensory cortices, and it enabled us to use the task also in people with possible or known problems in parts of the sensory cortices. If, for example, a person has face recognition dysfunction, data from the visual scene stimuli can still be used. As we aim to make this task suitable for large-scale population-based studies, which mostly examine people of different ages and health states, it can give usable and comparable information on a wide range of sensory and memory functions.

### Encoding Success Activity

The ESA maps demonstrated that the auditory cortex and visual cortex showed sensory-specific ESA (Figure 4). In contrast, the hippocampus and a subset of default network structures—including the precuneus and angular gyrus—showed ESA across both sensory domains. These results suggest that sensory-independent, or multimodal, brain regions form a core memory network (Johnson and Rugg, 2007; Kim et al., 2010; Gilmore et al., 2015; Kim, 2019). The precuneus showed negative ESA, consistent with previous findings on task-induced deactivation in the default network (Raichle et al., 2001; Daselaar et al., 2004; Huijbers et al., 2013; Krieger-Redwood et al., 2016). Especially negative ESA seems to be altered under the influence of early Alzheimer's disease pathology (Sperling et al., 2010; Ewers et al., 2011; Jagust, 2013; Fu et al., 2020). Although hit-rates for visual stimuli and especially for scene stimuli were slightly higher and might have resulted in an unbalanced weighting (compare Discussion - Behavior section), the consistency with previous results demonstrates that small differences in the weighting did not influence results strongly. Therefore, our paradigm might be an efficient alternative for clinical and population studies that are interested in the functional responses of the memory system. Further, the encoding of both auditory and visual information allows investigators to disentangle factors that influence sensory-specific responses vs. alterations to the core memory system. One idea could be that age-related hearing loss is likely to affect auditory ESA, glaucoma in the retina is likely to affect visual ESA, and Alzheimer's pathology might target ESA in the core memory system. Our task is properly designed to disentangle these peripheral changes in sensory systems from alterations to the core memory network.

### Sustained and Transient (De)Activations

The activation maps (task > rest) between sustained and transient activities show largely an overlap between activated regions indicating that sustained and transient activations co-occur simultaneously in sensory cortices (Visscher et al., 2003; Petersen and Dubis, 2012). However, deactivation maps (rest > task) show no overlap (Supplementary Figure A.2). Hence, we did not find any brain region—within or outside of the default network—that simultaneously showed sustained and transient deactivations. The lack of overlap between sustained and transient deactivations is not easily explained by overfitting or competition within the mixed model, as we did find overlap between sustained and transient activities. This is also confirmed by our comparisons of parallel mixed model with the block-only and event-only models (Supplementary Figure A.3). We also found that the majority of brain regions showed transient and not sustained deactivations. We interpret these findings in terms of task-intrusive and spontaneous thoughts (Weissman et al., 2006; Andrews-Hanna et al., 2010; Christoff et al., 2016). Task-induced deactivations are modulated by task demands (McKiernan et al., 2003) consistent with transient deactivation in response to the task. This would mean that the higher the task demands, the more deactivation will occur. The relative lack of brain regions that showed sustained deactivation suggests that a stable pattern of reduced activity is very rare, whereas event modeling gives more information about deactivation. This finding is also consistent with other mixed design studies that suggested mean activity is a relatively poor predictor of task performance (Garrett et al., 2014) because it disregards differences between stimuli and easily overestimates outlier. Finally, it is also possible that task-induced deactivations are modulated by, but not very tightly coupled to, stimulus onset. This would hinder the separation of sustained and transient deactivation. This last explanation is consistent with spontaneous thoughts (e.g., on the task instruction or other distractions coming from the situation in the scanner) that are partially restricted by the cognitive demands but not tightly coupled to stimulus onset.

### Reliability

As we did not have data to conduct a test–retest reliability across the complete task, we estimated the task reliability using the second task session as the retest session. ICC analysis for the behavioral overall and sensory-specific data according to Koo and Li (2016) showed moderate-to-good reliability. Stimulus-specific ICCs were slightly lower. However, the two task sessions were not identical, as the order of blocks was different and new stimuli of the same conditions were presented. Therefore, we expect to have underestimated the actual ICC values, and we consider the obtained values to be quite plausible. The poor-to-moderate reliability values in the stimulus conditions confirmed our decision to exclude the separate stimulus conditions from ESA analyses. Overall, the moderate-to-good behavioral reliability permits the application of this task as an fMRI paradigm.

Voxel-wise ICC analysis for fMRI data smoothed on group-level shows in general lower ICC values than the behavioral data. These values are similar to those of other studies that reported low or very heterogeneous ICC values for fMRI tasks (Bennett and Miller, 2010; Barch et al., 2013; Brandt et al., 2013; Elliott et al., 2020). fMRI reliability is influenced by many factors, including scanner noise, physiological noise, cognitive factors and processes, sample size, sample characteristics, and task characteristics (Bennett and Miller, 2010; Noble et al., 2020). We observed in most contrasts high ICC values in regions showing high activity (especially visual and, more specifically, in scene-related areas). This is not completely unexpected, as high fMRI activity can reduce the influence of errors, which results in a decreased within-participant variation, which then leads to an increase in ICC values (Bennett and Miller, 2010; Brandt et al., 2013). However, we observed also in some highly activated regions a fairly low ICC (e.g., hippocampus, precuneus, or superior temporal gyrus). This could be a property of the regions showing a greater variability in the hemodynamic response. In this case, low ICC represents a low congruence between the two halves of activity of the task within participants, although activation was commonly observed over the complete task. Still, we consider our reliability estimates as quite realistic and conclude that even with the parallel presentation, the reliability in comparison with other fMRI studies is not reduced.

## Limitations

Parallel mixed block/event-related fMRI designs measure transient and sustained information within a single, time-efficient paradigm that includes a large number of stimuli. However, this makes mixed designs very complex, and regressors are always affected by both events and blocks. Especially, events are always modeled on a changing baseline. This can make it difficult to interpret the comparison between transient and sustained effects.

Our study was sufficiently powered to calculate sensory-specific and overall ESA. However, we were not able to calculate stimulus-specific ESA within our sample. In the current study, we only included behavioral results from a small sample of older participants. Although their memory performance was slightly worse than that of participants in our younger sample, the hit-rates were close to 50%. Therefore, we would expect this task to be also applicable in older populations as well.

Finally, we would like to mention that we were able to estimate low-to-moderate reliability using a split-half reliability. These estimates are comparable with those of other fMRI tasks, but reliability should be kept in mind especially when analyzing smaller sample sizes (Turner et al., 2018; Bossier et al., 2020; Noble et al., 2020).

## Conclusions and Outlook

The presented parallel mixed design task paradigm enables efficient mapping of a versatile number of contrasts in limited time, making it attractive to acquire task-evoked fMRI in an epidemiological context for large-scale studies. The ability to map sensory activity as well as sensory-specific and unspecific ESA, and the ability to separate sustained and transient activities, can provide new insights into the dynamics of fMRI across the life span (Jimura and Braver, 2010; Petersen and Dubis, 2012). Currently, it remains unclear how multiple modifiable factors like lifestyle, education, or blood pressure, together with non-modifiable factors, like APOE status or gender, determine the brain's functional responses over age and to pathology. Only large-scale population studies that possess sufficient power to dissociate these factors can provide answers to these questions.

Besides these relevant questions, we would like to encourage future studies using the task to evaluate between-scanner reliability and to explore intra-individual variability in more depth. As the task is easily applicable, it does not require special scanning parameters and shows quite strong activation in the relevant brain regions relating to the main functions, we would not assume a huge loss of information if the data were collected with different scanners. As mentioned, the task requires only 10 min of fMRI scanning time. However, for generating ESA contrasts, an additional 10 to 19 min of (post-scan) recognition task needs to be performed. For many large-scale population studies, the limiting factor is the fMRI scanning time. The additional recognition task can be performed during other structural scans, which makes it user-friendly and cost-efficient. Previously, we demonstrated that the scan quality was not affected, or even benefitted, from performing a task inside the MRI (Huijbers et al., 2017). In case no other scans are needed, it is also possible to perform the recognition task outside the scanner, as long as the delay is constant for all participants.

## Data Availability Statement

The datasets presented in this article are not readily available because of data protection regulations. On the group-level we uploaded the T-maps of the contrasts described in the article under https://neurovault.org/collections/IABCOPVN/ (persistent identifier: https://identifiers.org/neurovault.collection:4413). All tools used for the post processing are open source and described in detail in section Materials and Methods - Behavioral analysis and Materials and Methods - Functional MRI preprocessing. Task scripts can be assessed under https://nextcloud.dzne.de/index.php/s/THxy2YkmJLzYN8c. Requests to access the datasets should be directed to the Rhineland Study's Data Use and Access committee, Prof. Dr. Monique M. B. Breteler, RS-DUAC@dzne.de.

## Ethics Statement

The studies involving human participants were reviewed and approved by the medical ethics committee of the Medical Faculty of the University of Bonn. The patients/participants provided their written informed consent to participate in this study.

## Author Contributions

MMB: conceptualization, methodology, software, formal analysis, investigation, writing–original draft, and visualization. KD: formal analysis and writing–review and editing. SH: methodology and writing–review and editing. MS: software and writing–review and editing. TS: software, validation, resources, and writing–review and editing. MMBB: conceptualization, resources, data curation, writing–review and editing, supervision, and funding acquisition. WH: conceptualization, methodology, software, validation, formal analysis, investigation, resources, data curation, writing–review and editing, visualization, supervision, and project administration. All authors contributed to the article and approved the submitted version.

## Conflict of Interest

The authors declare that the research was conducted in the absence of any commercial or financial relationships that could be construed as a potential conflict of interest.
