# Supplementary material for: A Functional MRI Paradigm for Efficient Mapping of Memory Encoding Across Sensory Conditions
Source: Front Hum Neurosci. 2021 Jan 21;14:591721. doi: 10.3389/fnhum.2020.591721 (PMC7859438; doi:10.3389/fnhum.2020.591721)
Supplement: Supplementary file 1 [file Data_Sheet_1.PDF]

Supplementary Material

to

"A functional MRI paradigm for efficient mapping of memory encoding across sensory conditions"

1 Behavioral results in older adults 2

2 Association between age, memory performance and response bias 3

3 Sustained and transient deactivation 4

4 Cluster peaks for all contrasts 4

5 Model comparison between mixed, block- and event-only modeling 15

6 Analysis of reliability 18

6.1 Behavioral reliability analysis 18

6.2 Reliability of sensory-specific and encoding success activity 20

7 References 27

1 Behavioral results in older adults

To demonstrate the feasibility of our paradigm across the lifespan, a behavioral follow-up study in 21 adults over 30 years was conducted. The subsequent memory test took between 10 and 19 minutes (min). The auditory retrieval took on average 7.83 min (*SD* = 1.48, range 6.83-13.78 min) and visual retrieval 4.34 min (*SD* = 0.76, range 3.15-6.10 min). The subsequent memory performance, as defined by the Hit- and FA-rates, are listed in Table A.1 including paired t-tests. For each sensory and stimuli condition, the Hit-rate was significantly greater than the False Alarm (FA)-rate. The differences between the Hit-and FA-rates indicate that participants were able to remember items in this category.

Table A.1 Memory performance

	Hit-rate		FA-rate		Hit-rate vs. FA-rate			*d'*				
	M	*SD*	*M*	*SD*	*t*	df	*p*	*M*	*SD*	*t*	df	*p*
Auditory	0.52	0.19	0.25	0.12	8.12	20	< 0.001	0.76	0.43			
Visual	0.62	0.11	0.18	0.12	13.77	20	< 0.001	1.33	0.49			
Auditory vs. Visual										5.48	20	<0.001
Environmental	0.49	0.16	0.21	0.10	8.29	20	< 0.001	0.83	0.44			
Vocal	0.55	0.23	0.29	0.17	6.82	20	< 0.001	0.70	0.59			
Environmental vs. Vocal										1.32	20	0.203
Face	0.58	0.14	0.22	0.16	9.80	20	< 0.001	1.06	0.51			
Scene	0.65	0.16	0.13	0.11	15.47	20	< 0.001	1.70	0.56			
Face vs. Scene										6.56	20	<0.001

Mean (*M*) and standard deviation (*SD*) for Hit-rate (percentage of correct old items), False Alarm (FA)-rate (percentage of incorrect new items) and d-prime (*d'*) (difference between z-standardized Hit- and FA-rate) (N = 21). Paired t-tests are used to depict differences between Hit- and FA-rate and between *d'* of sensory and stimuli conditions.
Note: df indicates the degrees of freedom, *t* indicated the t-value and *p* indicates the p-value representing the significance level.

Across all conditions we found a d-pime (*d'*) of 1.01 (*SD* = 0.38) and a response bias (*c*) of 0.32 (*SD* = 0.27) (auditory: *c* = 0.34 (*SD* = 0.43); visual: *c* = 0.36 (*SD* = 0.31)). The response bias indicated that participants were relatively conservative ($t(20) = 5.57$, $p < 0.001$) and thus more likely to rate items as “new”. Paired t-tests indicated that memory was better for visual items compared with auditory items and for scene images better than for face images, but no there was no difference in memory performance between environmental and vocal stimuli (Table A.1).

Separated for presentation condition and over sensory conditions subsequent memory results showed for auditory stimuli in the older sample *d'* values of $M_{isolated} = 0.80$ ($SD_{isolated} = 0.59$) and $M_{parallel} = 0.68$ ($SD_{parallel} = 0.43$) and for visual stimuli in the older sample $M_{isolated} = 1.51$ ($SD_{isolated} = 0.56$) and

$M_{parallel}$ = 1.16 ($SD_{parallel}$ = 0.52). Results of a ANOVA confirmed a better subsequent memory performance for visual than for auditory stimuli independent of the presentation condition (isolated/parallel) ($F(1,20) = 29.57$, $p < 0.001$) also for the older sample. Also the presentation conditions showed a main effect indicating better subsequent memory for stimuli presented in isolation independent of the sensory modality ($F(1,20) = 9.19$, $p = 0.007$)). In contrast to the younger sample we did not find an interaction in this small older sample between sensory modality and the presentation condition ($F(1,20) = 4.28$, $p = 0.052$).

2 Association between age, memory performance and response bias

Across the lifespan, we found a negative correlation between age and memory performance, as measured by *d'* ($r(79) = -0.31$, $p = 0.005$) (Figure A.1). Within the group of young adults between age 19 and 30 (n = 60), we did not find a correlation between age and *d'* ($r(58) = -0.08$, $p = 0.519$). In contrast, within the group of adults older than 30 (n = 21), we found a negative correlation with age and *d'* ($r(19) = -0.53$, $p = 0.012$). Together, these findings confirm that age, especially in late life, is negatively associated with memory performance (e.g. 1).

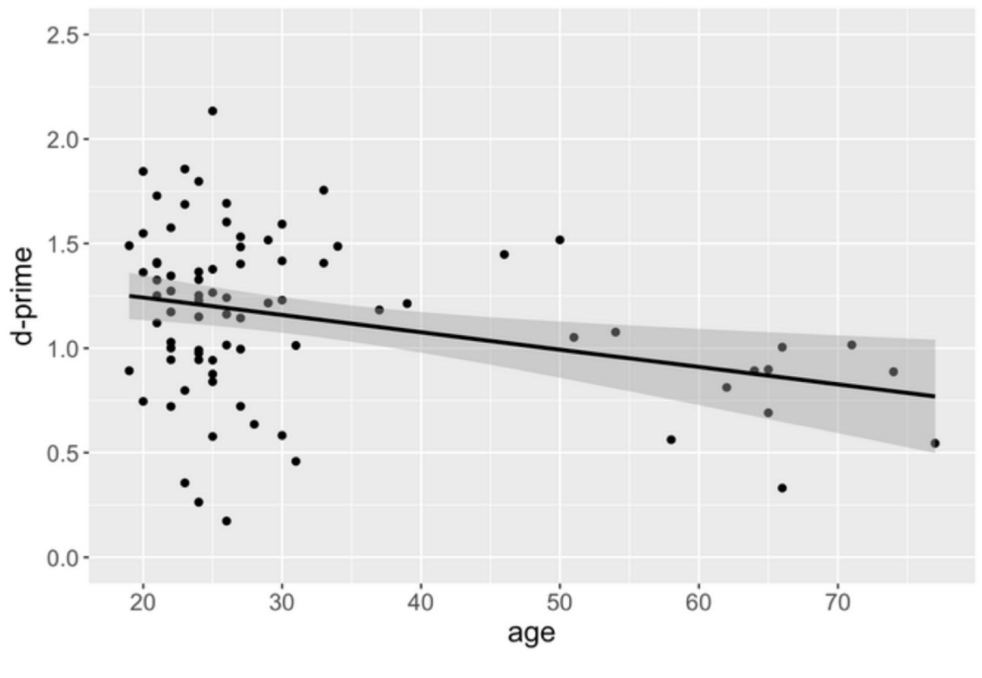

Figure A.1. Association between d-prime (z-standardized Hit-rate minus z-standardized FA-rate) and age (years).

Age and response bias were not correlated. Over all participants the correlation between age and response bias was $r(79) = -0.03$ ($p = 0.793$). Within the group of young adults between age 19 and 30 the correlation between age and response bias was $r(58) = 0.05$ ($p = 0.723$) and within the group of adults older than 30 the correlation between age and response bias was $r(19) = -0.06$, $p = 0.812$).

3 Sustained and transient deactivation

In addition to the pattern of sustained and transient activation in the manuscript we also examined the deactivation pattern. We mapped the block and event related activity from the mixed model analysis against the rest condition. For sustained auditory activity, we found the global minima in the right visual cortex (Figure A.2; c7). For the transient auditory activity, we found the minima in the right putamen (Figure A.2; c8). For sustained visual activity, we found the minima in the left temporoparietal junction (Figure A.2; c9). Finally, for transient visual activity, we found the minima in the left inferior temporal gyrus (Figure A.2; c10). For MNI coordinates and values see sustained (blocks) and transient (events) in manuscript Table 2.

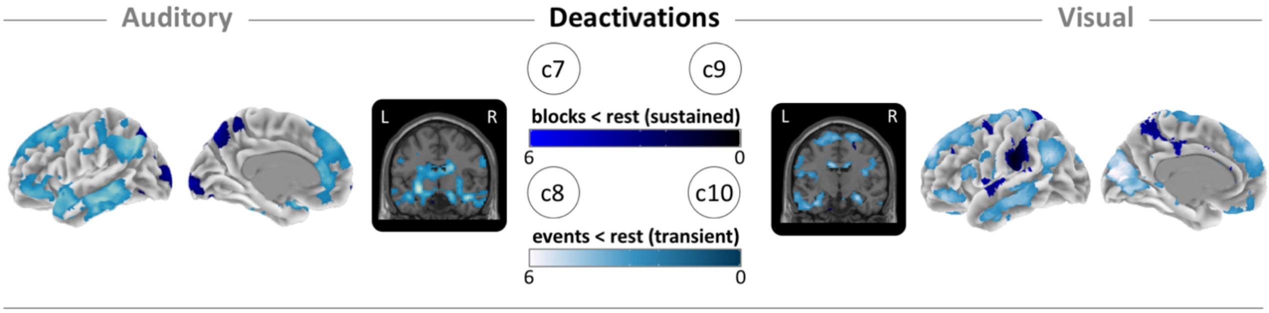

Figure A.2. Brain maps of block-related (sustained) and event-related (transient) deactivation. (c7) Auditory block versus rest deactivation (dark blue). (c8) Auditory event versus rest deactivation (light blue). (c9) Visual block versus rest deactivation (dark blue). (c10) Visual block versus rest deactivation (light blue). Brain activity is shown at a threshold of $p < 0.05$ (FDR-corrected) and the color intensity shows the t-value. Maps are uploaded under https://neurovault.org/collections/IABCOPVN/.

4 Cluster peaks for all contrasts

To elaborate on the contrast information, we included Table A.2 giving for all clusters with a minimum number of five voxels the cluster peak with the highest activation. All contrasts excluded activation in the cerebellum. As explained in the manuscript contrasts c2 and c3 were masked for auditory activity greater than visual activity (c1) and visual activity greater than auditory activity (c1) respectively. Clusters and cluster peaks were calculated using FIVE.m (https://habs.mgh.harvard.edu/researchers/data-tools/downloads/) an matlab integrated tool.

Table A.2 Cluster peaks for all contrasts

Contrast		Sensory-Specific Activity<br>Automatic Anatomical Labeling	Cluster Size	MNI (x,y,z)			t-value	BA
c1	Auditory > Visual	82 R. Superior Temporal Gyrus	9242	54	-1	-13	9.89	Right-BA22
		31 L. Anterior Cingulate Cortex	393	-9	35	5	3.86	Left-BA24
		89 L. Inferior Temporal Gyrus	46	-51	-34	-25	3.49	Left-BA20
		8 R. Middle Frontal Gyrus	38	27	20	35	2.97	Right-BA8
		7 L. Middle Frontal Gyrus	27	-27	17	38	2.94	Left-BA8
		58 R. Postcentral Gyrus	8	36	-43	71	2.88	Right-PrimSensory (1)
		32 R. Anterior Cingulate Cortex	10	6	17	29	2.72	Right-BA32
		11 L. Inferior Frontal Operculum	10	-39	11	23	2.39	Left-BA44
c1	Visual > Auditory	43 L. Calcarine Sulcus	10987	-3	-91	-4	13.13	Left-VisualAssoc (18)
		5 L. Superior Frontal Gyrus - Orbital Part	59	-9	68	-7	3.21	Left-BA10
		31 L. Anterior Cingulate Cortex	36	-3	5	26	3.17	Left-BA24
		3 L. Superior Frontal Gyrus	39	-9	62	35	3.15	Left-BA9
		4 R. Superior Frontal Gyrus	19	21	68	11	2.83	Right-BA10
		9 L. Middle Frontal Gyrus - Orbital Part	12	-36	56	-16	2.72	Left-BA10
		56 R. Fusiform Gyrus	6	27	-4	-43	2.70	Right-BA20
		5 L. Superior Frontal Gyrus - Orbital Part	8	-12	38	-25	2.66	Left-BA11
		56 R. Fusiform Gyrus	6	42	-13	-31	2.55	Right-BA20
		5 L. Superior Frontal Gyrus - Orbital Part	5	-18	50	-19	2.42	Left-BA11
c2	Environmental > Vocal	64 R. SupraMarginal Gyrus	158	57	-28	32	3.78	Right-BA40
		31 L. Anterior Cingulate Cortex	93	-6	35	8	3.54	Left-BA24
		30 R. Insula	119	39	14	-7	3.34	Right-Insula (13)
		29 L. Insula	27	-36	17	8	3.18	Left-BA45
		81 L. Superior Temporal Gyrus	32	-42	-16	-10	3.13	Left-BA22
		32 R. Anterior Cingulate Cortex	46	9	23	26	3.10	Right-BA32
		63 L. SupraMarginal Gyrus	42	-45	-37	29	3.07	Left-BA40
		34 R. Middle Cingulate Cortex	12	18	-31	41	2.71	Right-SensoryAssoc (5)

Contrast		Automatic Anatomical Labeling	Cluster Size	MNI (x,y,z)			t-value	BA
c2	Environmental > Vocal	82 R. Superior Temporal Gyrus	6	42	-22	2	2.64	Right-PrimAuditory (41)
c2	Vocal > Environmental	82 R. Superior Temporal Gyrus	8321	63	-1	-10	11.65	Right-BA22
		1 L. Precentral Gyrus	474	-54	-1	47	4.68	Left-BA6
		2 R. Precentral Gyrus	367	58	0	42	4.34	Right-BA6
		28 R. Gyrus Rectus	184	3	47	-16	4.25	Right-BA11
		37 L. Hippocampus	42	-18	-31	-1	3.62	Left-Thalamus (50)
		4 R. Superior Frontal Gyrus	52	15	-10	77	3.58	Right-BA6
		59 L. Superior Parietal Lobule	74	-24	-61	62	3.48	Left-BA7
		23 L. Superior Frontal Gyrus - Medial Part	65	-6	59	32	3.41	Left-BA9
		57 L. Postcentral Gyrus	30	-33	-31	71	3.38	Left-PrimSensory (1)
		2 R. Precentral Gyrus	43	45	-19	65	3.27	Right-BA6
		60 R. Superior Parietal Lobule	12	15	-79	56	3.20	Right-BA7
		60 R. Superior Parietal Lobule	69	24	-64	68	3.17	Right-BA7
		19 L. Supplementary Motor Area	30	-12	5	74	3.04	Left-BA6
		72 R. Caudate	21	15	-1	29	3.02	Right-BA24
		67 L. Precuneus	57	-6	-40	74	2.98	Left-SensoryAssoc (5)
		44 R. Calcarine Sulcus	5	27	-58	11	2.88	Right-BA23
		68 R. Precuneus	7	15	-52	23	2.84	Right-BA23
		4 R. Superior Frontal Gyrus	6	27	35	53	2.75	Right-BA8
		58 R. Postcentral Gyrus	10	21	-37	77	2.70	Right-SensoryAssoc (5)
		16 R. Inferior Frontal Gyrus - Orbital Part	7	54	32	-10	2.60	Right-BA47
		19 L. Supplementary Motor Area	7	-3	14	62	2.52	Left-BA6
c3	Face > Scene	55 L. Fusiform Gyrus	7350	-42	-49	-22	8.17	Left-Fusiform (37)
		28 R. Gyrus Rectus	235	6	50	-19	4.93	Right-BA11
		1 L. Precentral Gyrus	186	-30	-4	32	3.69	Left-BA6
		2 R. Precentral Gyrus	100	54	5	47	3.69	Right-BA6
		76 R. Pallidum	514	18	8	2	3.14	Right-Putamen (49)
		7 L. Middle Frontal Gyrus	87	-36	20	56	3.05	Left-BA8

Contrast		Automatic Anatomical Labeling	Cluster Size	MNI (x,y,z)			t-value	BA
c3	Face > Scene	23 L. Superior Frontal Gyrus - Medial Part	91	-6	62	32	3.02	Left-BA10
		36 R. Posterior Cingulate Cortex	36	15	-34	20	2.84	Right-Caudate (48)
		89 L. Inferior Temporal Gyrus	61	-33	-10	-43	2.76	Left-BA20
		7 L. Middle Frontal Gyrus	79	-39	41	35	2.44	Left-BA9
		87 L. Middle Temporal Pole	9	-39	17	-43	2.43	Left-BA38
		11 L. Inferior Frontal Operculum	31	-60	11	23	2.40	Left-BA44
		66 R. Angular Gyrus	6	45	-70	50	2.35	Right-BA39
		19 L. Supplementary Motor Area	6	-6	23	65	2.32	Left-BA6
		12 R. Inferior Frontal Operculum	11	27	2	32	2.20	Right-BA8
		48 R. Lingual Gyrus	8	3	-34	5	2.17	Right-Thalamus (50)
		1 L. Precentral Gyrus	5	-54	-4	50	2.17	Left-BA6
		57 L. Postcentral Gyrus	8	-66	-19	35	1.98	Left-PrimSensory (1)
		11 L. Inferior Frontal Operculum	5	-48	20	35	1.90	Left-BA8
c3	Scene > Face	55 L. Fusiform Gyrus	11769	-27	-49	-7	24.08	Left-BA19
		77 L. Thalamus	15	-15	-7	-1	3.18	Left-GlobPal (51)
		7 L. Middle Frontal Gyrus	14	-21	5	53	2.78	Left-BA6
		76 R. Pallidum	14	21	-7	-1	2.72	Right-GlobPal (51)
		39 L. Parahippocampal Gyrus	8	-6	5	-25	2.66	Left-Amygdala (53)
		26 R. Medial Surface of the Frontal Lobe - Orbital Part	5	12	59	-4	2.44	Right-BA10
		27 L. Gyrus Rectus	8	-9	23	-16	2.33	Left-BA11
		90 R. Inferior Temporal Gyrus	14	54	-55	-10	2.27	Right-Fusiform (37)
		9 L. Middle Frontal Gyrus - Orbital Part	10	-33	38	-13	2.24	Left-BA47
		71 L. Caudate	17	-6	17	-4	2.23	Left-Caudate (48)
		17 L. Rolandic Operculum	11	-42	-19	23	2.13	Left-PrimSensory (1)
		57 L. Postcentral Gyrus	15	-42	-28	56	2.10	Left-PrimSensory (1)

Encoding Success Activity (ESA) for isolated blocks

Contrast		Automatic Anatomical Labeling	Cluster Size	MNI (x,y,z)			t-value	BA
c4	positive ESA	40 R. Parahippocampal Gyrus	279	21	-7	-25	6.09	Right-Parahip (36)

Contrast		Automatic Anatomical Labeling	Cluster Size	MNI (x,y,z)			t-value	BA
c4	positive ESA	37 L. Hippocampus	336	-24	-10	-19	5.90	Left-Hippocampus (54)
		82 R. Superior Temporal Gyrus	182	45	-37	5	5.81	Right-BA21
		52 R. Middle Occipital Gyrus	456	33	-91	8	4.79	Right-VisualAssoc (18)
		15 L. Inferior Frontal Gyrus - Orbital Part	169	-39	32	-10	4.63	Left-BA47
		53 L. Inferior Occipital Gyrus	615	-27	-91	-7	4.53	Left-VisualAssoc (18)
		6 R. Superior Frontal Gyrus - Orbital Part	64	21	35	-13	4.51	Right-BA11
		85 L. Middle Temporal Gyrus	305	-60	-28	-1	4.42	Left-BA22
		28 R. Gyrus Rectus	221	6	44	-19	4.01	Right-BA11
		11 L. Inferior Frontal Operculum	102	-42	14	23	3.93	Left-BA44
		14 R. Inferior Frontal Gyrus - pars triangulars	112	45	35	2	3.80	Right-BA46
		82 R. Superior Temporal Gyrus	11	60	-1	-10	3.39	Right-BA22
		55 L. Fusiform Gyrus	6	-30	-1	-49	3.12	Left-BA38
		1 L. Precentral Gyrus	13	-54	-1	50	3.05	Left-BA6
		2 R. Precentral Gyrus	11	57	-1	50	2.99	Right-BA6
c4	negative ESA	68 R. Precuneus	2802	9	-70	44	-6.37	Right-BA7
		4 R. Superior Frontal Gyrus	1274	27	65	8	-5.37	Right-BA10
		7 L. Middle Frontal Gyrus	591	-30	38	38	-4.39	Left-BA9
		62 R. Inferior Parietal Lobule	757	54	-49	41	-4.32	Right-BA39
		72 R. Caudate	60	9	20	-4	-4.04	Right-Caudate (48)
		63 L. SupraMarginal Gyrus	451	-63	-37	41	-3.97	Left-BA40
		7 L. Middle Frontal Gyrus	183	-21	8	53	-3.79	Left-BA6
		30 R. Insula	39	36	11	11	-3.63	Right-BA44
		71 L. Caudate	66	-15	23	-4	-3.62	Left-Caudate (48)
		32 R. Anterior Cingulate Cortex	33	6	41	-1	-3.59	Right-BA32
		89 L. Inferior Temporal Gyrus	6	-57	-31	-31	-3.56	Left-BA20
		34 R. Middle Cingulate Cortex	149	6	38	32	-3.45	Right-BA8
		29 L. Insula	8	-36	8	11	-3.40	Left-BA44
		90 R. Inferior Temporal Gyrus	23	54	-19	-37	-3.39	Right-BA20

Contrast		Automatic Anatomical Labeling	Cluster Size	MNI (x,y,z)			t-value	BA
c4	negative ESA	85 L. Middle Temporal Gyrus	11	-63	-61	-4	-3.14	Left-Fusiform (37)
		80 R. Heschls Gyrus	25	51	-10	8	-3.14	Right-PrimAuditory (41)
		30 R. Insula	14	33	20	-19	-3.04	Right-BA47
		46 R. Cuneus	9	15	-82	26	-2.99	Right-BA19
		82 R. Superior Temporal Gyrus	6	45	-7	-7	-2.93	Right-Insula (13)
		71 L. Caudate	7	-9	-1	20	-2.87	Left-Caudate (48)
		57 L. Postcentral Gyrus	6	-21	-28	77	-2.73	Left-PrimSensory (1)
		7 L. Middle Frontal Gyrus	7	-45	20	47	-2.72	Left-BA8
		71 L. Caudate	5	-9	17	17	-2.57	Left-Caudate (48)
c5	positive auditory ESA	85 L. Middle Temporal Gyrus	709	-60	-13	-4	5.72	Left-BA22
		82 R. Superior Temporal Gyrus	635	57	-25	-1	5.63	Right-BA22
		15 L. Inferior Frontal Gyrus - Orbital Part	138	-36	35	-10	4.94	Left-BA47
		41 L. Amygdala	119	-33	2	-28	4.56	Left-BA38
		84 R. Superior Temporal Pole	29	36	5	-25	4.27	Right-BA38
		16 R. Inferior Frontal Gyrus - Orbital Part	51	48	32	-4	3.96	Right-BA47
		14 R. Inferior Frontal Gyrus - pars triangulars	38	39	20	20	3.79	Right-BA44
		6 R. Superior Frontal Gyrus - Orbital Part	23	24	32	-13	3.53	Right-BA47
		27 L. Gyrus Rectus	45	0	41	-22	3.51	Right-BA11
		38 R. Hippocampus	16	24	-7	-19	3.49	Right-Hippocampus (54)
		11 L. Inferior Frontal Operculum	25	-45	11	20	3.35	Left-BA44
		46 R. Cuneus	27	15	-103	8	3.24	Right-VisualAssoc (18)
		1 L. Precentral Gyrus	5	-54	-1	50	2.97	Left-BA6
		51 L. Middle Occipital Gyrus	5	-15	-106	-1	2.94	Left-VisualAssoc (18)
		39 L. Parahippocampal Gyrus	5	-12	-25	-11.5	2.86	Left-Parahip (36)
		51 L. Middle Occipital Gyrus	5	-30	-97	2	2.75	Left-VisualAssoc (18)
c5	negative auditory ESA	68 R. Precuneus	2421	21	-55	23	-5.06	Right-BA23
		8 R. Middle Frontal Gyrus	657	33	38	44	-4.77	Right-BA9
		7 L. Middle Frontal Gyrus	125	-21	2	50	-4.37	Left-BA6

Contrast		Automatic Anatomical Labeling	Cluster Size	MNI (x,y,z)			t-value	BA
c5	negative auditory ESA	5 L. Superior Frontal Gyrus - Orbital Part	113	-21	68	-4	-3.95	Left-BA10
		7 L. Middle Frontal Gyrus	114	-39	32	29	-3.81	Left-BA9
		56 R. Fusiform Gyrus	81	36	-37	-16	-3.61	Right-Fusiform (37)
		32 R. Anterior Cingulate Cortex	30	6	41	-1	-3.60	Right-BA32
		4 R. Superior Frontal Gyrus	99	30	62	5	-3.51	Right-BA10
		72 R. Caudate	15	9	20	-4	-3.48	Right-Caudate (48)
		51 L. Middle Occipital Gyrus	41	-36	-82	29	-3.28	Left-BA19
		52 R. Middle Occipital Gyrus	6	36	-70	2	-3.09	Right-BA19
		73 L. Putamen	11	-18	20	2	-3.04	Left-Caudate (48)
c6	positive visual ESA	54 R. Inferior Occipital Gyrus	551	27	-91	-4	5.31	Right-VisualAssoc (18)
		40 R. Parahippocampal Gyrus	112	21	-10	-25	5.21	Right-Parahip (36)
		51 L. Middle Occipital Gyrus	911	-36	-88	2	4.54	Left-VisualAssoc (18)
		56 R. Fusiform Gyrus	134	39	-31	-19	4.23	Right-Parahip (36)
		27 L. Gyrus Rectus	47	0	35	-25	3.68	Right-BA11
		82 R. Superior Temporal Gyrus	8	45	-37	5	3.56	Right-BA21
		1 L. Precentral Gyrus	9	-45	5	32	3.14	Left-BA6
c6	negative visual ESA	68 R. Precuneus	936	12	-67	32	-5.53	Right-BA7
		63 L. SupraMarginal Gyrus	200	-57	-25	23	-4.30	Left-BA40
		48 R. Lingual Gyrus	93	12	-79	-1	-4.17	Right-VisualAssoc (18)
		85 L. Middle Temporal Gyrus	27	-63	-61	-4	-4.12	Left-Fusiform (37)
		4 R. Superior Frontal Gyrus	58	27	65	11	-3.93	Right-BA10
		64 R. SupraMarginal Gyrus	22	69	-31	38	-3.59	Right-BA40
		72 R. Caudate	6	9	11	17	-3.47	Right-Caudate (48)
		5 L. Superior Frontal Gyrus - Orbital Part	8	-18	20	-16	-3.45	Left-BA11
		62 R. Inferior Parietal Lobule	69	60	-52	41	-3.43	Right-BA39
		8 R. Middle Frontal Gyrus	15	39	29	41	-3.37	Right-BA9
		5 L. Superior Frontal Gyrus - Orbital Part	18	-27	59	-4	-3.36	Left-BA10
		7 L. Middle Frontal Gyrus	6	-36	38	41	-3.34	Left-BA9

Contrast		Automatic Anatomical Labeling	Cluster Size	MNI (x,y,z)			t-value	BA
c6	negative visual ESA	10 R. Middle Frontal Gyrus - Orbital Part	7	33	50	-1	-3.21	Right-BA10
		71 L. Caudate	6	-9	8	20	-3.13	Left-Caudate (48)
		47 L. Lingual Gyrus	7	-6	-76	-4	-3.13	Left-VisualAssoc (18)

Sustained (blocks)

Contrast		Automatic Anatomical Labeling	Cluster Size	MNI (x,y,z)			t-value	BA
c7	Auditory > rest (activation)	82 R. Superior Temporal Gyrus	22827	54	2	-13	9.71	Right-BA22
		68 R. Precuneus	10	27	-46	17	2.78	Right-BA23
		2 R. Precentral Gyrus	8	42	-22	68	2.48	Right-BA6
		57 L. Postcentral Gyrus	11	-63	-13	32	2.30	Left-PrimMotor (4)
		27 L. Gyrus Rectus	5	-3	14	-25	2.27	Left-BA25
c7	Auditory < rest (deactivation)	46 R. Cuneus	3270	18	-100	8	5.39	Right-VisualAssoc (18)
		40 R. Parahippocampal Gyrus	55	36	-37	-10	3.59	Right-Parahip (36)
		26 R. Medial Surface of the Frontal Lobe - Orbital Part	42	9	71	-7	3.22	Right-BA10
		39 L. Parahippocampal Gyrus	28	-33	-43	-4	2.75	Left-Parahip (36)
		4 R. Superior Frontal Gyrus	25	18	71	8	2.70	Right-BA10
		53 L. Inferior Occipital Gyrus	11	-48	-82	-4	2.70	Left-BA19
		39 L. Parahippocampal Gyrus	5	-9	2	-34	2.31	Left-Parahip (36)
c9	Visual > rest (activation)	43 L. Calcarine Sulcus	12653	-6	-88	-1	14.81	Left-PrimVisual (17)
		15 L. Inferior Frontal Gyrus - Orbital Part	1160	-48	44	-13	7.25	Left-BA47
		8 R. Middle Frontal Gyrus	437	51	32	35	6.95	Right-BA9
		27 L. Gyrus Rectus	258	-3	53	-16	5.64	Left-BA11
		31 L. Anterior Cingulate Cortex	32	-3	5	26	4.71	Left-BA24
		58 R. Postcentral Gyrus	11	66	-1	23	4.16	Right-PrimMotor (4)
		14 R. Inferior Frontal Gyrus - pars triangulars	14	54	26	5	3.69	Right-BA45
		86 R. Middle Temporal Gyrus	9	48	-40	8	3.62	Right-BA22
		57 L. Postcentral Gyrus	11	-63	-7	26	3.53	Left-PrimMotor (4)
c9	Visual < rest (deactivation)	63 L. SupraMarginal Gyrus	3613	-63	-25	26	6.01	Left-BA40
		78 R. Thalamus	94	3	-25	-1	4.33	Right-Thalamus (50)

Contrast		Automatic Anatomical Labeling	Cluster Size	MNI (x,y,z)			t-value	BA
c9	Visual < rest (deactivation)	30 R. Insula	53	39	11	5	4.21	Right-BA44
		85 L. Middle Temporal Gyrus	22	-63	-61	-1	4.18	Left-Fusiform (37)
		8 R. Middle Frontal Gyrus	427	24	32	38	4.12	Right-BA8
		77 L. Thalamus	125	-12	-16	20	3.82	Left-Thalamus (50)
		66 R. Angular Gyrus	11	48	-76	37	3.79	Right-BA39
		68 R. Precuneus	38	27	-46	17	3.67	Right-BA23
		34 R. Middle Cingulate Cortex	96	3	11	35	3.65	Right-BA32
		3 L. Superior Frontal Gyrus	123	-21	29	32	3.53	Left-BA8
		67 L. Precuneus	40	-24	-49	17	3.50	Left-BA23
		20 R. Supplementary Motor Area	75	15	-1	65	3.39	Right-BA6
		31 L. Anterior Cingulate Cortex	32	-12	38	-1	3.14	Left-BA32
		71 L. Caudate	24	-15	20	8	3.12	Left-Caudate (48)
		22 R. Olfactory Sulcus	13	9	8	-16	3.05	Right-BA25
		68 R. Precuneus	29	18	-52	35	3.00	Right-BA31
		21 L. Olfactory Sulcus	10	-9	5	-16	2.92	Left-NucAccumb (52)
		89 L. Inferior Temporal Gyrus	5	-48	-31	-28	2.80	Left-BA20
		58 R. Postcentral Gyrus	29	30	-28	41	2.80	Right-PrimSensory (1)
		88 R. Middle Temporal Pole	10	42	11	-31	2.59	Right-BA38
		31 L. Anterior Cingulate Cortex	26	0	29	23	2.42	Right-BA32

		Transient (events)						
Contrast		Automatic Anatomical Labeling	Cluster Size	MNI (x,y,z)			t-value	BA
c8	Auditory > rest (activation)	81 L. Superior Temporal Gyrus	572	-42	-28	8	7.89	Left-PrimAuditory (41)
		80 R. Heschls Gyrus	423	48	-22	8	7.12	Right-PrimAuditory (41)
		46 R. Cuneus	1560	15	-103	8	6.45	Right-VisualAssoc (18)
c8	Auditory < rest (deactivation)	22 R. Olfactory Sulcus	16830	21	8	-13	7.10	Right-Putamen (49)
		1 L. Precentral Gyrus	20	-30	-13	44	2.83	Left-BA6
		33 L. Middle Cingulate Cortex	20	0	-22	41	2.74	Right-BA31
		28 R. Gyrus Rectus	5	9	26	-28	2.68	Right-BA11

Contrast		Automatic Anatomical Labeling	Cluster Size	MNI (x,y,z)			t-value	BA
c8	Auditory < rest (deactivation)	57 L. Postcentral Gyrus	13	-24	-31	71	2.63	Left-PrimSensory (1)
		2 R. Precentral Gyrus	11	21	-28	71	2.48	Right-PrimMotor (4)
c10	Visual > rest (activation)	56 R. Fusiform Gyrus	1061	30	-55	-13	8.79	Right-Fusiform (37)
		55 L. Fusiform Gyrus	962	-27	-52	-7	7.59	Left-BA19
		78 R. Thalamus	161	6	-25	-4	4.50	Right-Thalamus (50)
		2 R. Precentral Gyrus	120	45	2	32	4.43	Right-BA6
		62 R. Inferior Parietal Lobule	56	27	-49	50	3.92	Right-BA7
		1 L. Precentral Gyrus	61	-39	2	32	3.85	Left-BA6
		21 L. Olfactory Sulcus	11	-9	5	-16	3.42	Left-NucAccumb (52)
		19 L. Supplementary Motor Area	16	-9	14	47	3.33	Left-BA6
		61 L. Inferior Parietal Lobule	43	-24	-49	53	3.22	Left-BA7
		78 R. Thalamus	20	9	-10	17	3.17	Right-Thalamus (50)
		34 R. Middle Cingulate Cortex	13	12	20	35	3.08	Right-BA8
		34 R. Middle Cingulate Cortex	20	15	-37	44	3.06	Right-SensoryAssoc (5)
		22 R. Olfactory Sulcus	8	6	11	-16	2.97	Right-BA25
		1 L. Precentral Gyrus	10	-39	-4	47	2.93	Left-BA6
		33 L. Middle Cingulate Cortex	15	-18	-34	41	2.81	Left-BA31
		82 R. Superior Temporal Gyrus	7	69	-40	20	2.79	Right-BA22
		78 R. Thalamus	8	12	-13	2	2.65	Right-Thalamus (50)
		84 R. Superior Temporal Pole	11	57	8	-10	2.64	Right-BA38
		77 L. Thalamus	5	-9	-10	17	2.64	Left-Thalamus (50)
		10 R. Middle Frontal Gyrus - Orbital Part	6	30	38	-16	2.48	Right-BA47
c10	Visual < rest (deactivation)	67 L. Precuneus	7280	-30	-49	5	7.15	Left-BA30
		3 L. Superior Frontal Gyrus	4753	-12	56	32	6.38	Left-BA9
		66 R. Angular Gyrus	332	48	-58	29	4.75	Right-BA39
		86 R. Middle Temporal Gyrus	27	57	5	-31	3.60	Right-BA38
		30 R. Insula	18	39	5	-16	3.15	Right-Insula (13)
		5 L. Superior Frontal Gyrus - Orbital Part	6	-15	68	-10	2.92	Left-BA10

Contrast		Automatic Anatomical Labeling	Cluster Size	MNI (x,y,z)			t-value	BA
c10	Visual < rest (deactivation)	90 R. Inferior Temporal Gyrus	29	60	-16	-28	2.83	Right-BA21

Contrasts represent: c1: block-based contrast between auditory versus visual stimuli blocks, c2: event-related contrast between environmental versus vocal sounds, c3: event-related contrast between face versus scene images, c4: ESA for all (visual and auditory) hits versus all misses, c5: ESA for auditory hits versus auditory misses, c6: ESA for visual hits versus visual misses, c7: auditory versus rest blocks, c9: visual versus rest blocks, c8: auditory events versus rest and c10: visual events versus rest. All brain regions are described with MNI coordinates (MNI(x,y,z)), t-values of the beta coefficients and the relating Brodmann-Area (BA). Contrast maps are uploaded under https://neurovault.org/collections/IABCOPVN/.

5 Model comparison between mixed, block- and event-only modeling

Our task was designed as a mixed model and therefore we analyzed it accordingly (one model including regressors for both blocks and events). Nevertheless, one can discuss whether results differ between different models and if yes in which direction and how much. To explore differences between models/designs we modeled our data additionally using a block design (block-only) and using an event design (event-only).

The block-only model contained two regressors: one for the auditory blocks and one for the visual blocks, identical to those in the mixed-design (Manuscript Figure 2). This allowed for estimation of the two block-related (sustained) contrasts: the auditory blocks versus rest (c7) and the visual blocks versus rest (c9) without the influence of the event-regressors. The event-only model contained eight regressors: one for each of the stimulus conditions (environmental, vocal, face and scene) by subsequent memory (hits and misses), identical to those in the mixed-design (Manuscript Figure 2). This allowed for estimation of the event-related (transient) contrasts without the influence of the block-regressors.

We extracted the fMRI activity, using a 5mm radius sphere, from four regions of interest (ROI) defined by the global maxima (positive ESA) and minima (negative ESA) of sensory-unspecific ESA (c4), and the maxima of auditory ESA (c5) and the maxima of visual ESA (c6) to compare sustained and transient activity and to compare the mixed, isolated block and isolated event models (Figure A.3, Table A.3).

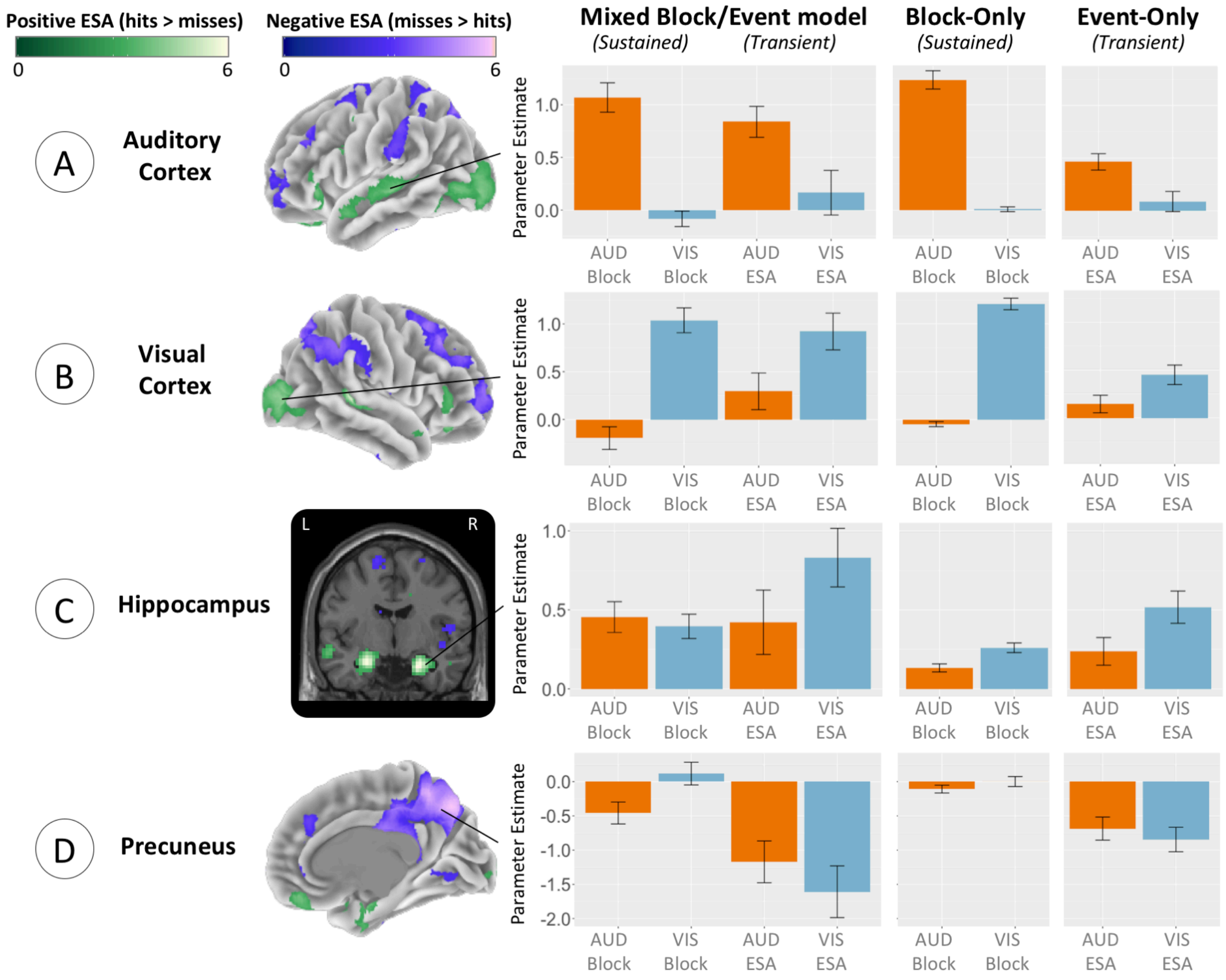

Figure A.3. Sustained (block) activity in contrast to rest activity, calculated with the mixed block/event design and the block-only design for auditory (AUD) and visual (VIS) stimuli separately for four regions of interest (ROIs). Transient (event) activity is shown as the difference between hit > miss (encoding success activity (ESA)) activity, calculated from the mixed block/event design and the event-only design for auditory and visual stimuli separately for four ROIs (A to D). ROIs were chosen using the ESA contrast (Manuscript Figure 5).

Table A.3 Model comparison of sustained and transient activity

**(A) Auditory cortex:** MNI$_{(x,y,z)}$ = [-60,-13,-4]							
Contrast		*M*	*SD*	*t*-value	*M*	*SD*	*t*-value
		Mixed Model			Block-only Model		
c7	Auditory blocks > rest	1.07	1.08	7.66	1.24	0.68	14.15
c9	Visual blocks > rest	-0.08	0.57	-1.14	0.01	0.18	0.33
c1	Auditory blocks > Visual blocks	1.15	1.34	6.66	1.23	0.70	13.52
		Mixed Model			Event-only Model		
c5	Auditory ESA (hits > misses)	0.84	1.14	5.69	0.46	0.60	5.97
c6	Visual ESA (hits > misses)	0.16	1.64	0.76	0.09	0.74	0.89
**(B) Visual cortex:** MNI$_{(x,y,z)}$ = [27,-91,-4]							
Contrast		*M*	*SD*	*t-value*	*M*	*SD*	*t*-value
		Mixed Model			Block-only Model		
c7	Auditory blocks > rest	-0.20	0.92	-1.64	-0.05	0.20	-1.88
c9	Visual blocks > rest	1.04	1.01	7.94	1.21	0.47	19.85
c1	Auditory blocks > Visual blocks	-1.23	1.37	-6.99	-1.26	0.55	-17.60
		Mixed Model			Event-only Model		
c5	Auditory ESA (hits > misses)	0.29	1.49	1.53	0.15	0.71	1.58
c6	Visual ESA (hits > misses)	0.92	1.49	4.70	0.45	0.79	4.32
**(C) Hippocampus:** MNI$_{(x,y,z)}$ = [21,-7,-25]							
Contrast		*M*	*SD*	*t*-value	*M*	*SD*	*t*-value
		Mixed Model			Block-only Model		
c7	Auditory blocks > rest	0.45	0.75	4.68	0.13	0.20	5.01
c9	Visual blocks > rest	0.40	0.60	5.13	0.26	0.23	8.58
c1	Auditory blocks > Visual blocks	0.06	0.96	0.47	-0.13	0.34	-2.95
		Mixed Model			Event-only Model		
c5	Auditory ESA (hits > misses)	0.42	1.58	2.07	0.24	0.68	2.72
c6	Visual ESA (hits > misses)	0.83	1.43	4.42	0.52	0.79	4.98
**(D) Precuneus:** MNI$_{(x,y,z)}$ = [9,-70,44]							
Contrast		*M*	*SD*	*t*-value	*M*	*SD*	*t*-value
		Mixed Model			Block-only Model		
c7	Auditory blocks > rest	-0.46	1.23	-2.87	-0.11	0.44	-1.91
c9	Visual blocks > rest	0.12	1.28	0.71	0.00	0.56	0.02
c1	Auditory blocks > Visual blocks	-0.57	1.94	-2.30	-0.11	0.66	-1.28
		Mixed Model			Event-only Model		
c5	Auditory ESA (hits > misses)	-1.17	2.37	-3.83	-0.69	1.31	-4.07
c6	Visual ESA (hits > misses)	-1.61	2.93	-4.19	-0.84	1.38	-4.65

Mean (*M*), standard deviation (*SD*) and *t*-values of beta coefficients from block and event-based encoding success activity (ESA) contrasts extracted from the mixed, the block-only and the event-only model for the four regions of interest.

For both the auditory and the visual cortex (A and B in Figure A.3, Table A.3) we found increased sustained activity for corresponding blocks (auditory and visual) coincided with the transient auditory ESA, using the mixed design, but not for the respective other sensory condition. In the hippocampus (C in Figure A.3, Table A.3), we found sustained activity for both auditory and visual blocks and this coincided with transient ESA for both sensory conditions, using the mixed design. In the precuneus (D in Figure A.3, Table A.3), we found slight sustained deactivations for the auditory block, but not for the visual block and this coincided with transient negative ESA for both sensory conditions, using the mixed design. For all four ROI's we found a consistent pattern of activity in the block-only and event-only model. Together, these analyses indicate that the mixed model identifies activity patterns relatively similar to more sparse models that include only block or event regressors. We also found slight differences in both the auditory and the visual cortex where activity seemed to be an additive combination of transient and sustained increases. For example, the auditory cortex showed both sustained and transient increases in auditory activity and the mixed model separates both contributions. In the hippocampus and precuneus the pattern is non-additive, which suggests that the sustained and transient response diverge. The hippocampus showed greater increases in activity in the mixed-model and the precuneus shows greater decreases. The hippocampus showed further sustained increases that coincide with relative transient decreases. Similarly, the precuneus showed sustained decreases that coincide with transient increases in activity.

6 Analysis of reliability

For our reliability analysis we split the task into two sessions (Blocks 1 to 16 and Blocks 17 to 32). Both sessions contained two blocks of each stimulus category presented isolated and one block for each possible parallel combination (vocal-face; vocal-scene; environmental-face; environmental-scene) presented parallel (Figure 1.).

6.1 Behavioral reliability analysis

For a first glance on the differences in performance of participants between the sessions we compared Hit-rate and FA-rate (Figure A.4). Overall and in both sensory conditions Hit-rates between the sessions differ between 13% and 17%, with the second session showing lower Hit-rates. This is very likely due to the task length, which seems to lead over time to a lower memory performance for participants. Nevertheless, our aim to create Hit-rates around 50% was met in both sessions. FA-rates did not differ between the two sessions, which shows equally well performance in rejecting new stimuli. The combination of Hit- and FA-rate (d-prime) confirms that memory performance was not equally well between the two sessions, but with values between 0.89 and 2.08 (Table A.4) we measured memory performance in both sessions and not random answering.

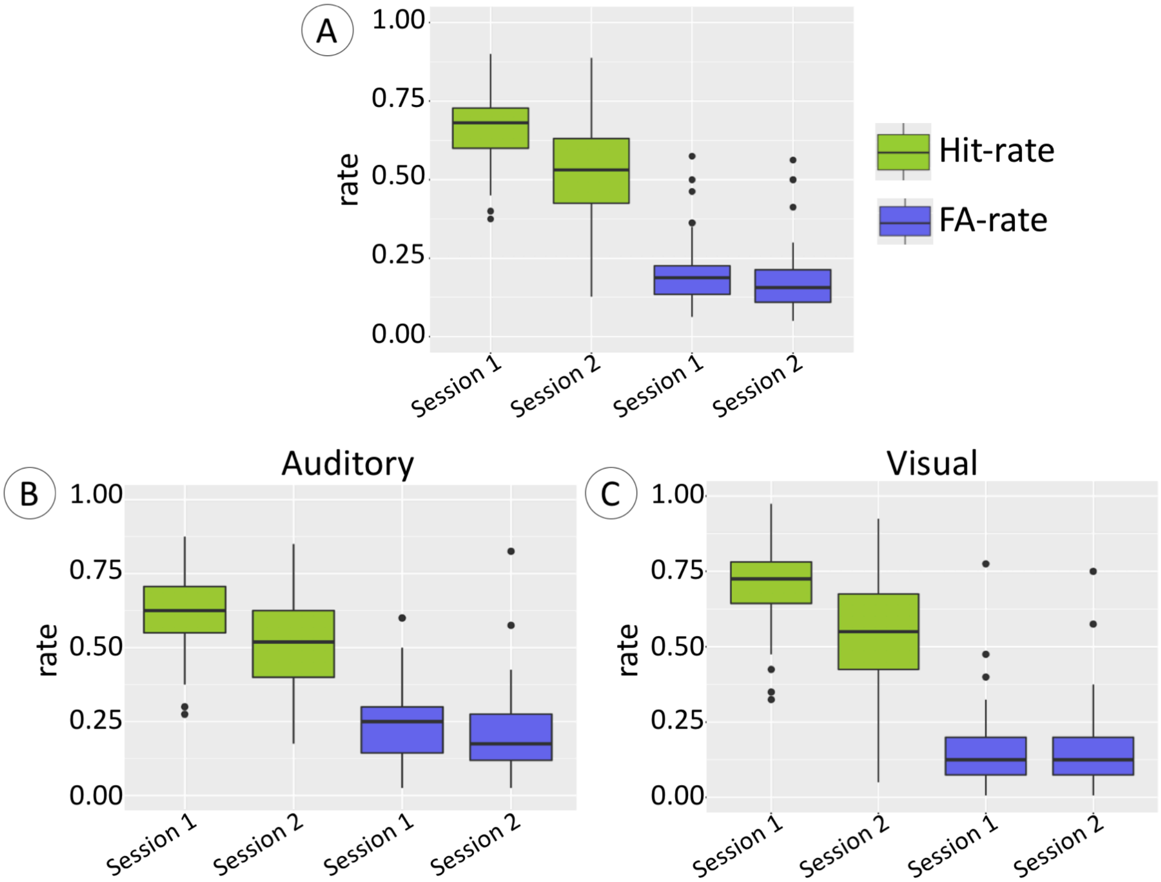

Figure A.4. Difference of Hit- and FA-rate between the two task sessions. (A) Hit-and FA-rate over all stimuli conditions. (B) Hit- and FA-rate for all auditory stimuli. (C) Hit- and FA-rate for all visual stimuli.

Table A.4 shows the behavioral ICC values, their confidence intervals and the *F*-statistics.

Table A.4 Intraclass correlation coefficients for behavioral analysis

		Session 1 Mean (SD)	Session 2 Mean (SD)	Intraclass Correlation	95% CI		F-Test with true value 0; df1 = 59, df2 = 59	
					LB	UB	Value	*p*
**Hit-rate**		0.67 (0.11)	0.52 (0.14)	.655	.483	.779	4.81	.000
	Auditory	0.63 (0.14)	0.50 (0.16)	.704	.549	.812	5.75	.000
	Environmental	0.60 (0.17)	0.44 (0.16)	.557	.355	.710	3.52	.000
	Vocal	0.66 (0.16)	0.57 (0.19)	.618	.434	.753	4.24	.000
	Visual	0.71 (0.13)	0.54 (0.19)	.551	.347	.705	3.45	.000
	Face	0.69 (0.16)	0.49 (0.23)	.400	.165	.593	2.34	.001
	Scene	0.73 (0.16)	0.60 (0.19)	.511	.297	.676	3.09	.000
**FA-rate**		0.20 (0.10)	0.18 (0.11)	.812	.705	.884	9.66	.000
	Auditory	0.24 (0.13)	0.21 (0.14)	.766	.637	.853	7.54	.000
	Environmental	0.20 (0.11)	0.18 (0.14)	.587	.393	.731	3.84	.000
	Vocal	0.29 (0.17)	0.24 (0.16)	.738	.597	.835	6.64	.000
	Visual	0.15 (0.12)	0.15 (0.13)	.739	.599	.836	6.68	.000
	Face	0.20 (0.15)	0.19 (0.16)	.723	.576	.825	6.22	.000
	Scene	0.11 (0.11)	0.11 (0.13)	.549	.345	.704	3.44	.000
d'		1.36 (0.41)	1.05 (0.47)	.675	.510	.792	5.16	.000
	Auditory	1.11 (0.54)	0.90 (0.52)	.622	.439	.756	4.29	.000
	Environmental	1.20 (0.61)	0.89 (0.66)	.461	.237	.639	2.71	.000
	Vocal	1.10 (0.66)	1.00 (0.56)	.435	.205	.619	2.54	.000
	Visual	1.73 (0.63)	1.31 (0.68)	.649	.475	.775	4.70	.000
	Face	1.51 (0.71)	0.99 (0.77)	.499	.282	.667	2.99	.000
	Scene	2.08 (0.76)	1.73 (0.87)	.557	.354	.709	3.51	.000

Intraclass correlation coefficients (ICC) separately for Hit-, FA-rate and d-prime (*d'*) tested against the null hypothesis. A two-way model using single units of each participant was applied to assess the consistency between the outcomes of the two sessions.
Note: CI indicates the confidence interval with LB (lower bound) and UB (upper bound).

6.2 Reliability of sensory-specific and encoding success activity

Voxel-wise ICC analysis for the fMRI data is based on the slice time corrected and normalized, but non-smoothed data. Figure A.5 shows the smoothed results from the ICC analysis in comparison to the results for the task-based activity analyses for the four main contrasts auditory vs. visual (c1), environmental vs. vocal (c2), face vs. scene (c3) and hits vs. misses (encoding success activity, c4). Table A.5 shows the smoothed ICC values for the ROIs defined above (see "Model comparison between mixed, block- and event-only modeling"). Table A.6 shows median, minimum and maximum ICC values for each cluster defined in the previous section ("Cluster peaks for all contrasts").

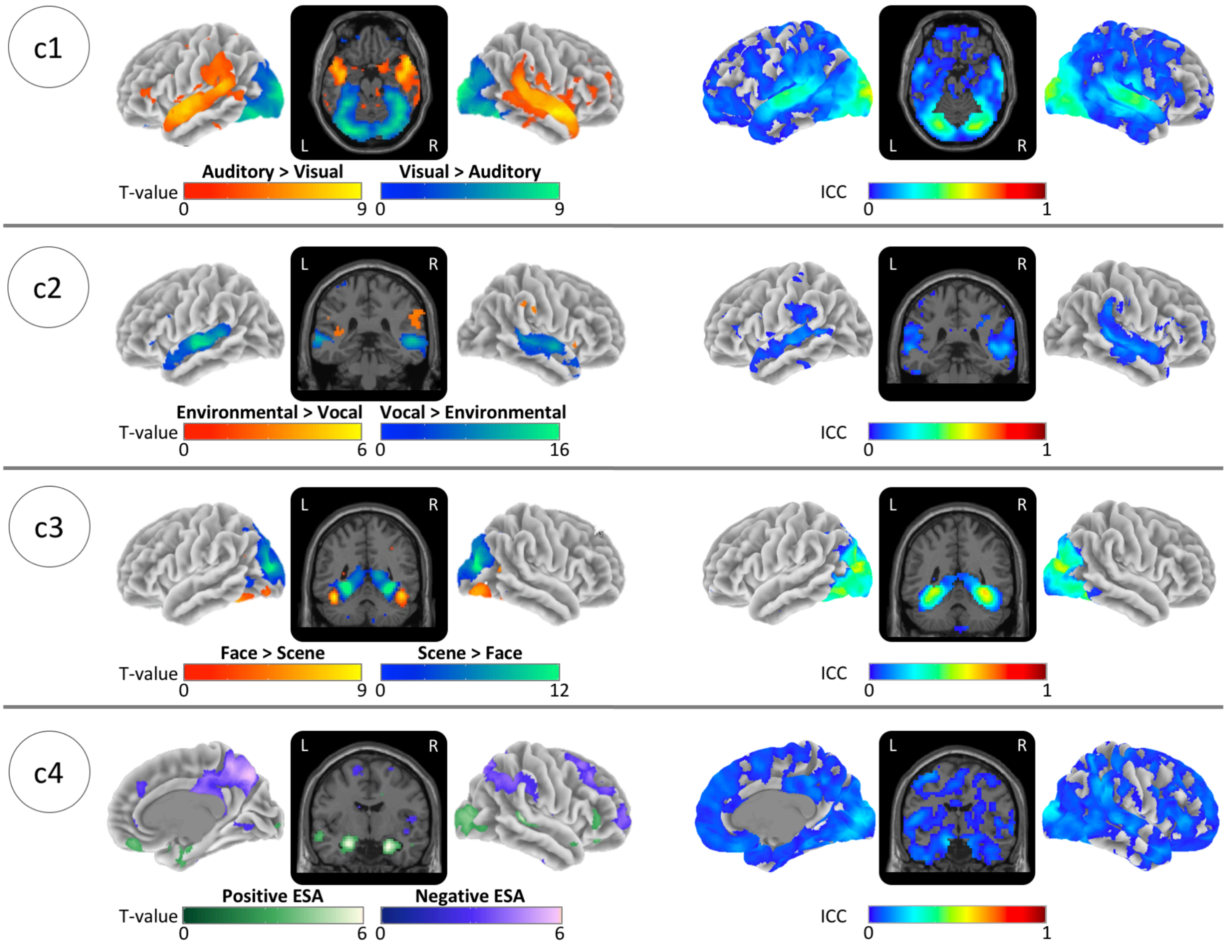

Figure A.5. ICC results in comparison to the task-based activity for the contrasts c1 (visual vs. auditory), c2 (environmental vs. vocal), c3 (face vs. scene) and c4 (encoding success activity (ESA), hits vs. misses). The left column shows brain activity at a threshold of p < 0.05 (FDR-corrected). The right column shows ICCs between 0 and 1. Grey areas represent ICC values under 0, which are considered as poor agreement similarly to 0 (2). Contrast c2 was masked by auditory greater visual activity ($p < 0.05$ FDR, see c1 left column), and contrast c3 was masked by visual greater auditory activity ($p < 0.05$ FDR, see c1 left column). Intraclass correlation coefficient maps as well as contrast maps are uploaded under https://neurovault.org/collections/IABCOPVN/.

Table A.5 Intraclass correlation coefficients for ROIs

Contrast		Region	MNI$_{(x,y,z)}$			Intraclass Correlation	BA
		**Sensory–Specific Activity**					
c1	Auditory > Visual	Auditory cortex	54,	- 1,	-13	.227	22/41
	Visual > Auditory	Visual cortex	-3,	-91,	-4	.380	17/18
c2	Environmental > Vocal	Temporoparietal junction	57,	-28,	32	.026	39/40
	Vocal > Environmental	Superior temporal gyrus	63,	-1,	-10	.044	22
c3	Face > Scene	Fusiform gyrus	-42,	-49,	-22	.388	37
	Scene > Face	Parahippocampal gyrus	-27,	-49,	-7	.514	19/36
		**Encoding success activity (ESA)**					
Contrast		Region	MNI$_{(x,y,z)}$			Intraclass Correlation	BA
c4	Positive ESA	Hippocampus	21,	-7,	-25	.067	36/54
	Negative ESA	Precuneus	9,	-70,	44	.024	7

Intraclass correlation coefficients for the main activated Regions (ROIs) of the contrasts Auditory vs. Visual stimuli (c1), Environmental vs. Vocal stimuli (c2), Face vs. Scene stimuli (c3) and Hit vs. Miss stimuli (c4). All brain regions are described with MNI coordinates (MNI$_{(x,y,z)}$) and the relating Brodmann-Area (BA). Intraclass correlation coefficient maps as well as contrast maps are uploaded under https://neurovault.org/collections/IABCOPVN/.

Table A.6 Median intraclass correlation coefficients for clusters

Contrast		Sensory-specific contrasts<br>Intraclass correlation coefficient<br>Median	Min	Max	Cluster size	Cluster activation peaks<br>MNI $_{(x,y,z)}$			Automatic Anatomical Labeling	BA
c1	Auditory > Visual	0.060	0.000	0.449	9242	54	-1	-13	82 R. Superior Temporal Gyrus	Right-BA22
		0.007	0.000	0.093	393	-9	35	5	31 L. Anterior Cingulate Cortex	Left-BA24
		0.000	0.000	0.067	46	-51	-34	-25	89 L. Inferior Temporal Gyrus	Left-BA20
		0.017	0.000	0.095	38	27	20	35	8 R. Middle Frontal Gyrus	Right-BA8
		0.052	0.000	0.104	27	-27	17	38	7 L. Middle Frontal Gyrus	Left-BA8
		0.008	0.000	0.056	8	36	-43	71	58 R. Postcentral Gyrus	Right-PrimSensory (1)
		0.004	0.000	0.016	10	6	17	29	32 R. Anterior Cingulate Cortex	Right-BA32
		0.072	0.058	0.104	10	-39	11	23	11 L. Inferior Frontal Operculum	Left-BA44
c1	Visual > Auditory	0.203	0.000	0.664	10987	-3	-91	-4	43 L. Calcarine Sulcus	Left-VisualAssoc (18)
		0.005	0.000	0.061	59	-9	68	-7	5 L. Superior Frontal Gyrus - Orbital Part	Left-BA10
		0.000	0.000	0.017	36	-3	5	26	31 L. Anterior Cingulate Cortex	Left-BA24
		0.030	0.000	0.072	39	-9	62	35	3 L. Superior Frontal Gyrus	Left-BA9
		0.026	0.000	0.041	19	21	68	11	4 R. Superior Frontal Gyrus	Right-BA10
		0.077	0.039	0.126	12	-36	56	-16	9 L. Middle Frontal Gyrus - Orbital Part	Left-BA10
		0.000	0.000	0.001	6	27	-4	-43	56 R. Fusiform Gyrus	Right-BA20
		0.004	0.000	0.020	8	-12	38	-25	5 L. Superior Frontal Gyrus - Orbital Part	Left-BA11
		0.000	0.000	0.009	6	42	-13	-31	56 R. Fusiform Gyrus	Right-BA20
		0.072	0.047	0.104	5	-18	50	-19	5 L. Superior Frontal Gyrus - Orbital Part	Left-BA11
c2	Environmental > Vocal	0.035	0.000	0.118	158	57	-28	32	64 R. SupraMarginal Gyrus	Right-BA40
		0.000	0.000	0.016	93	-6	35	8	31 L. Anterior Cingulate Cortex	Left-BA24
		0.000	0.000	0.083	119	39	14	-7	30 R. Insula	Right-Insula (13)
		0.014	0.000	0.074	27	-36	17	8	29 L. Insula	Left-BA45
		0.000	0.000	0.000	32	-42	-16	-10	81 L. Superior Temporal Gyrus	Left-BA22
		0.000	0.000	0.004	46	9	23	26	32 R. Anterior Cingulate Cortex	Right-BA32
		0.000	0.000	0.069	42	-45	-37	29	63 L. SupraMarginal Gyrus	Left-BA40
		0.000	0.000	0.032	12	18	-31	41	34 R. Middle Cingulate Cortex	Right-SensoryAssoc (5)
		0.000	0.000	0.000	6	42	-22	2	82 R. Superior Temporal Gyrus	Right-PrimAuditory (41)
c2	Vocal > Environmental	0.022	0.000	0.206	8321	63	-1	-10	82 R. Superior Temporal Gyrus	Right-BA22
		0.000	0.000	0.067	474	-54	-1	47	1 L. Precentral Gyrus	Left-BA6
		0.000	0.000	0.143	367	58	0	42	2 R. Precentral Gyrus	Right-BA6
		0.000	0.000	0.000	184	3	47	-16	28 R. Gyrus Rectus	Right-BA11
		0.000	0.000	0.058	42	-18	-31	-1	37 L. Hippocampus	Left-Thalamus (50)
		0.020	0.000	0.077	52	15	-10	77	4 R. Superior Frontal Gyrus	Right-BA6
		0.000	0.000	0.065	74	-24	-61	62	59 L. Superior Parietal Lobule	Left-BA7

Contrast		Intraclass correlation coefficient			Cluster size	Cluster activation peaks				
		Median	Min	Max		MNI $_{(x,y,z)}$			Automatic Anatomical Labeling	BA
c2	Vocal > Environmental	0.000	0.000	0.021	65	-6	59	32	23 L. Superior Frontal Gyrus - Medial Part	Left-BA9
		0.019	0.000	0.063	30	-33	-31	71	57 L. Postcentral Gyrus	Left-PrimSensory (1)
		0.000	0.000	0.008	43	45	-19	65	2 R. Precentral Gyrus	Right-BA6
		0.015	0.000	0.054	12	15	-79	56	60 R. Superior Parietal Lobule	Right-BA7
		0.000	0.000	0.036	69	24	-64	68	60 R. Superior Parietal Lobule	Right-BA7
		0.000	0.000	0.028	30	-12	5	74	19 L. Supplementary Motor Area	Left-BA6
		0.000	0.000	0.000	21	15	-1	29	72 R. Caudate	Right-BA24
		0.000	0.000	0.029	57	-6	-40	74	67 L. Precuneus	Left-SensoryAssoc (5)
		0.000	0.000	0.000	5	27	-58	11	44 R. Calcarine Sulcus	Right-BA23
		0.000	0.000	0.012	7	15	-52	23	68 R. Precuneus	Right-BA23
		0.000	0.000	0.000	6	27	35	53	4 R. Superior Frontal Gyrus	Right-BA8
		0.000	0.000	0.000	10	21	-37	77	58 R. Postcentral Gyrus	Right-SensoryAssoc (5)
		0.000	0.000	0.045	7	54	32	-10	16 R. Inferior Frontal Gyrus - Orbital Part	Right-BA47
		0.000	0.000	0.006	7	-3	14	62	19 L. Supplementary Motor Area	Left-BA6
c3	Face > Scene	0.013	0.000	0.548	7350	-42	-49	-22	55 L. Fusiform Gyrus	Left-Fusiform (37)
		0.011	0.000	0.078	235	6	50	-19	28 R. Gyrus Rectus	Right-BA11
		0.000	0.000	0.095	186	-30	-4	32	1 L. Precentral Gyrus	Left-BA6
		0.000	0.000	0.108	100	54	5	47	2 R. Precentral Gyrus	Right-BA6
		0.003	0.000	0.160	514	18	8	2	76 R. Pallidum	Right-Putamen (49)
		0.000	0.000	0.042	87	-36	20	56	7 L. Middle Frontal Gyrus	Left-BA8
		0.000	0.000	0.031	91	-6	62	32	23 L. Superior Frontal Gyrus - Medial Part	Left-BA10
		0.008	0.000	0.031	36	15	-34	20	36 R. Posterior Cingulate Cortex	Right-Caudate (48)
		0.022	0.000	0.064	61	-33	-10	-43	89 L. Inferior Temporal Gyrus	Left-BA20
		0.000	0.000	0.006	79	-39	41	35	7 L. Middle Frontal Gyrus	Left-BA9
		0.013	0.005	0.036	9	-39	17	-43	87 L. Middle Temporal Pole	Left-BA38
		0.032	0.000	0.073	31	-60	11	23	11 L. Inferior Frontal Operculum	Left-BA44
		0.000	0.000	0.000	6	45	-70	50	66 R. Angular Gyrus	Right-BA39
		0.000	0.000	0.000	6	-6	23	65	19 L. Supplementary Motor Area	Left-BA6
		0.009	0.000	0.042	11	27	2	32	12 R. Inferior Frontal Operculum	Right-BA8
		0.046	0.007	0.068	8	3	-34	5	48 R. Lingual Gyrus	Right-Thalamus (50)
		0.000	0.000	0.000	5	-54	-4	50	1 L. Precentral Gyrus	Left-BA6
		0.000	0.000	0.019	8	-66	-19	35	57 L. Postcentral Gyrus	Left-PrimSensory (1)
		0.000	0.000	0.009	5	-48	20	35	11 L. Inferior Frontal Operculum	Left-BA8
c3	Scene > Face	0.133	0.000	0.608	11769	-27	-49	-7	55 L. Fusiform Gyrus	Left-BA19
		0.006	0.000	0.029	15	-15	-7	-1	77 L. Thalamus	Left-GlobPal (51)
		0.089	0.000	0.149	14	-21	5	53	7 L. Middle Frontal Gyrus	Left-BA6
		0.000	0.000	0.000	14	21	-7	-1	76 R. Pallidum	Right-GlobPal (51)

Contrast		Intraclass correlation coefficient			Cluster size	Cluster activation peaks				
		Median	Min	Max		MNI (x,y,z)			Automatic Anatomical Labeling	BA
c3	Scene > Face	0.034	0.018	0.042	8	-6	5	-25	39 L. Parahippocampal Gyrus	Left-Amygdala (53)
		0.048	0.024	0.058	5	12	59	-4	26 R. Medial Surface of the Frontal Lobe - Orbital Part	Right-BA10
		0.001	0.000	0.017	8	-9	23	-16	27 L. Gyrus Rectus	Left-BA11
		0.108	0.000	0.234	14	54	-55	-10	90 R. Inferior Temporal Gyrus	Right-Fusiform (37)
		0.000	0.000	0.012	10	-33	38	-13	9 L. Middle Frontal Gyrus - Orbital Part	Left-BA47
		0.039	0.005	0.106	17	-6	17	-4	71 L. Caudate	Left-Caudate (48)
		0.000	0.000	0.000	11	-42	-19	23	17 L. Rolandic Operculum	Left-PrimSensory (1)
		0.000	0.000	0.045	15	-42	-28	56	57 L. Postcentral Gyrus	Left-PrimSensory (1)
		**Encoding Success Activity (ESA) contrasts for isolated blocks**								
Contrast		Intraclass correlation coefficient			Cluster size	Cluster activation peaks				
		Median	Min	Max		MNI (x,y,z)			Automatic Anatomical Labeling	BA
c4	positive ESA	0.043	0.000	0.155	279	21	-7	-25	40 R. Parahippocampal Gyrus	Right-Parahip (36)
		0.052	0.000	0.197	336	-24	-10	-19	37 L. Hippocampus	Left-Hippocampus (54)
		0.055	0.000	0.169	182	45	-37	5	82 R. Superior Temporal Gyrus	Right-BA21
		0.108	0.000	0.279	456	33	-91	8	52 R. Middle Occipital Gyrus	Right-VisualAssoc (18)
		0.041	0.000	0.150	169	-39	32	-10	15 L. Inferior Frontal Gyrus - Orbital Part	Left-BA47
		0.096	0.000	0.242	615	-27	-91	-7	53 L. Inferior Occipital Gyrus	Left-VisualAssoc (18)
		0.017	0.000	0.071	64	21	35	-13	6 R. Superior Frontal Gyrus - Orbital Part	Right-BA11
		0.039	0.000	0.146	305	-60	-28	-1	85 L. Middle Temporal Gyrus	Left-BA22
		0.066	0.000	0.151	221	6	44	-19	28 R. Gyrus Rectus	Right-BA11
		0.013	0.000	0.094	102	-42	14	23	11 L. Inferior Frontal Operculum	Left-BA44
		0.080	0.000	0.160	112	45	35	2	14 R. Inferior Frontal Gyrus - pars triangulars	Right-BA46
		0.016	0.000	0.049	11	60	-1	-10	82 R. Superior Temporal Gyrus	Right-BA22
		0.000	0.000	0.000	6	-30	-1	-49	55 L. Fusiform Gyrus	Left-BA38
		0.064	0.035	0.113	13	-54	-1	50	1 L. Precentral Gyrus	Left-BA6
		0.000	0.000	0.000	11	57	-1	50	2 R. Precentral Gyrus	Right-BA6
c4	negative ESA	0.050	0.000	0.198	2802	9	-70	44	68 R. Precuneus	Right-BA7
		0.029	0.000	0.177	1274	27	65	8	4 R. Superior Frontal Gyrus	Right-BA10
		0.041	0.000	0.166	591	-30	38	38	7 L. Middle Frontal Gyrus	Left-BA9
		0.024	0.000	0.201	757	54	-49	41	62 R. Inferior Parietal Lobule	Right-BA39
		0.001	0.000	0.051	60	9	20	-4	72 R. Caudate	Right-Caudate (48)
		0.015	0.000	0.170	451	-63	-37	41	63 L. SupraMarginal Gyrus	Left-BA40
		0.000	0.000	0.062	183	-21	8	53	7 L. Middle Frontal Gyrus	Left-BA6
		0.000	0.000	0.025	39	36	11	11	30 R. Insula	Right-BA44
		0.025	0.000	0.068	66	-15	23	-4	71 L. Caudate	Left-Caudate (48)
		0.000	0.000	0.048	33	6	41	-1	32 R. Anterior Cingulate Cortex	Right-BA32
		0.000	0.000	0.021	6	-57	-31	-31	89 L. Inferior Temporal Gyrus	Left-BA20

Contrast		Intraclass correlation coefficient			Cluster size	Cluster activation peaks				
		Median	Min	Max		MNI (x,y,z)			Automatic Anatomical Labeling	BA
c4	negative ESA	0.053	0.000	0.103	149	6	38	32	34 R. Middle Cingulate Cortex	Right-BA8
		0.000	0.000	0.015	8	-36	8	11	29 L. Insula	Left-BA44
		0.008	0.000	0.046	23	54	-19	-37	90 R. Inferior Temporal Gyrus	Right-BA20
		0.051	0.028	0.108	11	-63	-61	-4	85 L. Middle Temporal Gyrus	Left-Fusiform (37)
		0.031	0.000	0.092	25	51	-10	8	80 R. Heschls Gyrus	Right-PrimAuditory (41)
		0.028	0.000	0.078	14	33	20	-19	30 R. Insula	Right-BA47
		0.045	0.020	0.073	9	15	-82	26	46 R. Cuneus	Right-BA19
		0.000	0.000	0.000	6	45	-7	-7	82 R. Superior Temporal Gyrus	Right-Insula (13)
		0.000	0.000	0.000	7	-9	-1	20	71 L. Caudate	Left-Caudate (48)
		0.000	0.000	0.000	6	-21	-28	77	57 L. Postcentral Gyrus	Left-PrimSensory (1)
		0.072	0.052	0.099	7	-45	20	47	7 L. Middle Frontal Gyrus	Left-BA8
		0.024	0.000	0.042	5	-9	17	17	71 L. Caudate	Left-Caudate (48)

Contrasts represent: c1: block-based contrast between auditory versus visual stimuli blocks, c2: event-related contrast between environmental versus vocal sounds, c3: event-related contrast between face versus scene images, c4: ESA for all (visual and auditory) hits versus all misses, c5: ESA for auditory hits versus auditory misses, c6: ESA for visual hits versus visual misses, c7: auditory versus rest blocks, c9: visual versus rest blocks, c8: auditory events versus rest and c10: visual events versus rest. For all found clusters, median, min and max of intraclass coefficients are shown and linked to the cluster activation peaks preseted in Table A.2. All cluster activation peaks are described with MNI coordinates (MNI(x,y,z)) and the relating Brodmann-Area (BA). Intraclass correlation coefficient maps as well as contrast maps are uploaded under https://neurovault.org/collections/IABCOPVN/.

7 References

1. Nyberg L, Lövdén M, Riklund K, Lindenberger U, Bäckman L. Memory aging and brain maintenance. Trends Cogn Sci [Internet]. 2012;16(5):292–305. Available from: https://doi.org/10.1016/j.tics.2012.04.005

2. Giraudeau B. Negative values of the intraclass correlation coefficient are not theoretically possible. J Clin Epidemiol [Internet]. 1996;49(10):1205–6. Available from: https://doi.org/10.1016/0895-4356(96)00053-4
